# Evaluating the evidence-based potential of six large language models in paediatric dentistry: a comparative study on generative artificial intelligence

**DOI:** 10.1007/s40368-025-01012-x

**Published:** 2025-02-22

**Authors:** Anastasia Dermata, Aristidis Arhakis, Miltiadis A. Makrygiannakis, Kostis Giannakopoulos, Eleftherios G. Kaklamanos

**Affiliations:** 1 School of Dentistry, Aristotle University of Thessaloniki, 54124 Thessaloniki, Greece; 2 School of Dentistry, National and Kapodistrian University of Athens, 11527 Athens, Greece; 3 School of Dentistry, European University Cyprus, 2404 Nicosia, Cyprus; 4 Hamdan Bin Mohammed College of Dental Medicine (HBMCDM), Mohammed Bin Rashid University of Medicine and Health Sciences (MBRU), Dubai, UAE

**Keywords:** Paediatric dentistry, Large language models, Google gemini, OpenAI’s, ChatGPT, Microsoft’s copilot

## Abstract

**Purpose:**

The use of large language models (LLMs) in generative artificial intelligence (AI) is rapidly increasing in dentistry. However, their reliability is yet to be fully founded. This study aims to evaluate the diagnostic accuracy, clinical applicability, and patient education potential of LLMs in paediatric dentistry, by evaluating the responses of six LLMs: Google AI’s Gemini and Gemini Advanced, OpenAI’s ChatGPT-3.5, -4o and -4, and Microsoft’s Copilot.

**Methods:**

Ten open-type clinical questions, relevant to paediatric dentistry were posed to the LLMs. The responses were graded by two independent evaluators from 0 to 10 using a detailed rubric**.** After 4 weeks, answers were reevaluated to assess intra-evaluator reliability. Statistical comparisons used Friedman’s and Wilcoxon’s and Kruskal–Wallis tests to assess the model that provided the most comprehensive, accurate, explicit and relevant answers.

**Results:**

Variations of results were noted. Chat GPT 4 answers were scored as the best (average score 8.08), followed by the answers of Gemini Advanced (8.06), ChatGPT 4o (8.01), ChatGPT 3.5 (7.61), Gemini (7,32) and Copilot (5.41). Statistical analysis revealed that Chat GPT 4 outperformed all other LLMs, and the difference was statistically significant. Despite variations and different responses to the same queries, remarkable similarities were observed. Except for Copilot, all chatbots managed to achieve a score level above 6.5 on all queries.

**Conclusion:**

This study demonstrates the potential use of language models (LLMs) in supporting evidence-based paediatric dentistry. Nevertheless, they cannot be regarded as completely trustworthy. Dental professionals should critically use AI models as supportive tools and not as a substitute of overall scientific knowledge and critical thinking.

**Supplementary Information:**

The online version contains supplementary material available at 10.1007/s40368-025-01012-x.

## Introduction

Like most other scientific areas, paediatric dentistry greatly benefits from the innovations of generative artificial intelligence (AI) technologies. Large language models, also known as LLMs, mainly belong to a category of deep learning architectures known as transformer networks (Eggmann 2023). They constitute neural networks that can learn context and meaning by identifying relationships in sequential data, such as the words in a sentence (Eggmann 2023). They emerged recently online, and their popularity was immediate. In fact, within 3 months from its launch at the end of 2022, OpenAI’s ChatGPT hit a milestone of 100 million users, reflecting the tremendous speed at which such technologies are being put into practice today. To form coherent and relevant dialogues, ChatGPT includes a huge dataset from Wikipedia, books, and online articles (Vaishya 2023; Sallam 2023; Fergus 2023). These models are based on advanced techniques that use sophisticated algorithms and complex neural network, including Self-Attention and Positional Encoding. This gives the feel for fine details in the text, allowing them to come up with appropriate solutions and answers to very intricate questions in clinical settings (Brynjolfsson 2023).

Despite their short course of existence, development of LLMs has shown a continuous evolution, as witnessed by an increase in their complexity and a growing user base. Following the release of the OpenAI’s ChatGPT, large technology brands, Microsoft and Google announced and produced competitor LLMs: namely Bing Chat and Bard. Currently, ChatGPT 4.5 is available, and Bing Chat and Bard have been replaced by Microsoft Copilot and Google Gemini, respectively. With these novel tools. new scenarios for AI use are possible. For instance, Gemini deploys more sophisticated algorithms and makes use of a much larger dataset, thereby significantly improving the quality and variety of interactions the user may experience (Schade 2023). Other projects like GitHub Copilot further underline the prevalent surge in the LLM domain. These tools provide decision-making support—making it into coding assistance—and they are applied across a range of fields, including professional dental practice settings.

In recent years, there has been a significant increase in AI applications and tools within the area of dentistry. The main goal of integrating AI into this field is to assist professionals in providing enhanced healthcare services. It was highlighted by a recent white paper that these tools can reinforce several functions, including image analysis, radiograph interpretation, using neural networks for diagnoses, data synthesis, material information, clinical techniques for improving outcomes, patient record management, and applications in forensic dentistry, orthodontics, periodontology, endodontics, caries diagnosis, treatment planning, and even enhancing communication with patients (Vaishya 2023). Beyond its use in other dental specialities, the use of LLMs could be helpful in the field of paediatric dentistry, but also in the development of better educational schemes and information sharing to benefit paediatric patients. It offers instant integration of patient data with the latest clinical research, enabling health professionals to stay updated on new treatment trends and techniques from reliable sources. In this sense, the process of developing the tool would support evidence-based practices that, via accuracy and patient-centred care, would later follow forth (Islam 2022, Carrillo-Perez 2022, Seah 2023).

Given the increasing reliance on AI tools in healthcare, evaluating their role in paediatric dentistry is timely and crucial. In this study, we evaluated the diagnostic accuracy, clinical applicability, and potential utility of six large language models (LLMs)—Google AI’s Gemini and Gemini Advanced, OpenAI’s ChatGPT-3.5, -4o, and -4, and Microsoft’s Copilot—in paediatric dentistry. By assessing their responses to open-ended clinical questions, this study aims to identify their strengths, limitations, and potential applications in supporting evidence-based dental care.

## Methods

### Study design

This paper aims to comparatively assess response effectiveness and accuracy of selected generative artificial intelligence large language models in paediatric dentistry. The clinical objectives of the study are: to determine to what extent these models handle the clinical questions common in paediatric dental practice and to compare model responses with established scientific criteria.

This paper has a prospective study design. All clinically relevant questions were asked to six different large language models.

Selection of large language models (LLMs).

The LLMs chosen for this study are among the most advanced and widely used in the field of AI conversational systems. The following were included:

1. *Google’s Gemini—*known for its robust data processing capabilities.
2. *Google’s Gemini Advanced—*an enhanced version of Gemini, offering more refined algorithmic interactions.
3. *OpenAI’s ChatGPT-3.5—*a widely accessible model renowned for its deep learning efficiency.
4. *OpenAI’s ChatGPT-4—*an advanced version of ChatGPT, known for its improved contextual understanding and accuracy.
5. *OpenAI’s ChatGPT*-4o—a faster version of ChatGPT-4.
6. *Microsoft’s Copilo—*utilizes advanced AI to assist in a variety of professional tasks, including dental decision support.

Each question was drafted with appropriate clinical terminology in the context of paediatric dentistry and was designed to be open ended, thus assuring that the response would be in written form (Supplementary Table 1). The questions were grounded in contemporary evidence-based guidelines and literature, focusing on real-world clinical scenarios commonly encountered in paediatric dentistry, such as the management of early childhood caries, dental trauma, behaviour guidance, and treatment planning for young patients. This approach ensured that the questions reflected both the complexity and practical relevance of paediatric dental practice, enabling a robust evaluation of the LLMs’ capabilities in handling clinically pertinent issues. In an effort to simulate usage in the real world where the paediatric dentist may call on consultation in a spontaneous, on-demand fashion, each LLM was queried once for each question by one of the authors without an opportunity for the consultant to ask “clarifying” or “elicitation” questions or seek “re-wording” of the question. This method provided a response from each LLM under similar controlled conditions, demonstrating their ability to deliver immediate and precisely relevant answers to paediatric dental issues. Also, no LLMs’ training had taken place previously for us to assess their original potential to answer these questions.

To avoid bias from repetitive interaction or varying interpretations by different essayists, each question was asked only once and was not revisited by a different researcher. This approach really focused on the efficiency of the LLMs to deliver timely and relevant information, something very important in the clinical environment when working at a fast pace, where time and accuracy become paramount.

Interaction was limited, in this study, to a single query per LLM without an opportunity for follow-up questions or clarifications or elaboration. The focus was specifically on the ability of the LLMs to retrieve directly relevant answers for complex clinical questions. This represented a one-shot, lean querying approach, to extract the most informative responses from any LLM for real-time clinical decisions compared to the time-consuming nature of a more traditional multi-interaction evaluation process.

### Evaluation process

#### Evaluators and assessment methodology

For this study, two examiners who are paediatric dentistry specialists and who have been extensively involved in the undergraduate and postgraduate training of dental professionals were selected. One examiner has a PhD and holds a faculty position at a university, and another is a PhD student in paediatric dentistry. While the study utilized two evaluators, their specialized knowledge provided robust and reliable evaluations. Furthermore, the use of a detailed scoring rubric and intra-evaluator reliability assessment minimized subjectivity and ensured consistency in the scoring process.

Each of the LLM responses was rated individually by the evaluators. The responses were blinded to ensure objectivity; in this case, each LLM received a letter code and no evaluator knew which model they were rating at any point in time. Blind assessment was undertaken to ensure that no bias occurred towards any LLM.

#### Rating and scoring rubric

Responses were graded from 0 (minimum) to 10 (maximum) using an extremely detailed rubric, which was designed for this study (Supplementary Table 2). The rubric was devised with respect to how precisely, relevantly, clearly, and completely each answer was from the paediatric dentistry perspective. The "criterion standard" for each answer was based on evidence and clinical guidelines that were supported by science and was previously decided upon and scored a 10. It was compared with the scoring that the LLMs’ answers received.

#### Re-scoring for reliability

The second round of marking was conducted 4 weeks later to test the intra-evaluator reliability. This re-evaluation identified consistency in the scoring decisions for every evaluator, hence further improving the reliability of the assessment tool.

This rigorous evaluation methodology will guarantee that the findings from a study into AI effectiveness in paediatric dental care are robust and replicable, with clear insights into how each LLM does really meet the needs of paediatric dental professionals in real-world scenarios.

#### Statistical analysis

The data was summarized by calculating indices of central tendency (mean and median values), and indices of variability (minimum and maximum values, standard deviations, standard errors of mean values, and coefficient of variation). To test inter-evaluator reliability, i.e. if there is a correlation between the grades of the evaluators, r and rho were calculated. To test reliability, Cronbach’s alpha and intraclass correlation coefficient were calculated. Furthermore, to test the differences between the grades, Friedman’s, Kruskal–Wallis and Wilcoxon’s tests were performed. All statistical analyses were performed with the IBM SPSS v.29.0 enhanced with the module Exact Tests (for performing the Monte Carlo simulation method). The significance level in all hypothesis and testing procedures was predetermined at *a* = 0.05 (*p* ≤ 0.05) (Mehta and Patel 1996).

## Results

Table 1 presents the descriptive statistics for the scores given by the two evaluators to the answers provided by the six LLMs on two different occasions, 4 weeks apart. In both evaluations, LLM Chat GPT 4 received the highest scores and LLM COPILOT received the lowest scores.

**Table 1:** Descriptive statistics for the scores given by the two evaluators to the answers provided by the six LLMs on two different occasions, 4 weeks apart

Score 1												
	Copilot		ChatGPT 4o		Gemini Advanced		ChatGPT 4		Gemini		ChatGPT 3.5	
Evaluator	1	2	1	2	1	2	1	2	1	2	1	2
Min	2.50	4.25	6.50	7.00	6.50	7.50	6.50	7.25	5.50	6.25	6.00	7.00
Median	5.30	6.25	7.80	8.88	7.80	8.13	7.60	8.38	7.00	7.75	7.30	7.75
Max	5.75	7.00	8.50	9.50	9.25	9.50	9.25	9.50	8.25	9.00	8.75	9.25
Mean	4.87	5.80	7.60	8.45	7.83	8.33	7.83	8.35	6.98	7.63	7.20	7.95
SEM	0.31	0.31	0.18	0.28	0.27	0.27	0.24	0.30	0.24	0.25	0.26	0.25
SD	0.99	0.99	0.56	0.90	0.86	0.72	0.76	0.94	0.76	0.80	0.82	0.79
CoV	20.3%	17.1%	7.4%	10.7%	11.0%	8.6%	9.7%	11.3%	10.9%	10.5%	11.4%	9.9%

Score 2												
	Copilot		ChatGPT 4o		Gemini Advanced		ChatGPT 4		Gemini		ChatGPT 3.5	
Evaluator	1	2	1	2	1	2	1	2	1	2	1	2
Min	3.25	4.25	6.5	7.00	7.0	7.50	6.5	7.25	5.75	6.25	6.25	7.25
Median	5.25	6.50	7.75	8.88	7.63	8.13	7.75	8.38	7.0	7.75	7.13	7.75
Max	5.75	7.25	8.25	9.50	9.0	9.50	8.75	9.50	8.25	9.00	8.75	9.25
Mean	5.00	5.95	7.53	8.45	7.78	8.30	7.80	8.33	7.05	7.63	7.28	8.00
SEM	0.23	0.32	0.17	0.28	0.23	0.23	0.19	0.29	0.24	0.25	0.23	0.24
SD	0.74	1.02	0.53	0.90	0.72	0.73	0.60	0.92	0.75	0.79	0.71	0.76
CoV	14.2%	17.1%	7.0%	10.6%	9.3%	8.8%	7.7%	11.0%	10.6%	10.4%	9.8%	9.5%

*CoV* coefficient of variance, *Max* maximum, *Min* minimum, *SD* standard deviation, *SEM* standard error of mean

The inter-evaluator reliability, i.e. the correlation between the scores given by the two evaluators is presented in Table 2. Both Pearson’s *r* and Spearman’s *rho* tests showed strong and statistically significant correlations, indicating that the two evaluators graded the answers of the six LLMs similarly.

**Table 2:** Correlation between the scores given by the two evaluators to the answers provided by the six LLMs on two different occasions, 4 weeks apart. Statistically significant values in bold

LLM [Evaluator 1-2]	Score 1		Score 2	
	*r* (*p* value)	*rho* (*p* value)	*r* (*p* value)	*rho* (*p* value)
Copilot	0.965 **(< 0.001)**	0.885 **(0.001)**	0.992 **(< 0.001)**	0.949 **(< 0.001)**
ChatGPT 4o	0.950 **(0.001)**	0.964 **(< 0.001)**	0.838 **(0.002)**	0.804 **(0.004)**
Gemini Advanced	0.881 **(0.001)**	0.876 **(0.001)**	0.863 **(0.001)**	0.814 **(0.004)**
ChatGPT 4	0.965 **(< 0.001)**	0.942 **(< 0.001)**	0.683 **(0.040)**	0.634 **(0.042)**
Gemini	0.794 **(0.006)**	0.874 **(0.001)**	0.896 **(< 0.001)**	0.997 **(< 0.001)**
ChatGPT 3.5	0.973 **(< 0.001)**	0.950 **(< 0.001)**	0.728 **(0.017)**	0.828 **(0.003)**

Cronbach’s alpha and the intraclass correlation coefficient, both indicators of reliability, were high in this study. All Cronbach’s alpha values exceeded 0.6 and all intraclass correlation coefficients were statistically significant (Table 3). This finding was supported by Friedman’s and Wilcoxon tests, which found that there was not overall statistically significant difference in the scores given by the two evaluators to the answers of the LLMs on both occasions (Table 4).

**Table 3:** Cronbach’s *a* and intraclass correlation coefficient [ICC] for the scores given by the two evaluators to the answers provided by the six LLMs on two different occasions, 4 weeks apart. Statistically significant values in bold

LLM	Score 1			Score 2			Pooled scores 1 and 2		
	Cronbach’s *a*	ICC (*p* value)		Cronbach’s *a*	ICC (*p* value)		Cronbach’s *a*)	ICC (*p* value)	
		Single	Average		Single	Average		Single	Average
Copilot	0.961	0.924 **(< 0.001)**	0.961 **(< 0.001)**	0.993	0.967 **(< 0.001)**	0.983 **(< 0.001)**	0.892	0.673 **(< 0.001)**	0.892 **(< 0.001)**
ChatGPT 4o	0.988	0.975 **(< 0.001)**	0.988 **(< 0.001)**	0.996	0.992 **(< 0.001)**	0.996 **(< 0.001)**	0.858	0.601 **(< 0.001)**	0.858 **(< 0.001)**
Gemini Advanced	0.979	0.958 **(< 0.001)**	0.979 **(< 0.001)**	0.997	0.994 **(< 0.001)**	0.997 **(< 0.001)**	0.974	0.902 **(< 0.001)**	0.974 **(< 0.001)**
ChatGPT 4	0.966	0.933 **(< 0.001)**	0.966**(< 0.001)**	0.998	0.996 **(< 0.001)**	0.998 **(< 0.001)**	0.867	0.621 **(< 0.001)**	0.867 **(< 0.001)**
Gemini	0.987	0.975 **(< 0.001)**	0.987 **(< 0.001)**	0.995	0.989 **(< 0.001)**	0.995 **(< 0.001)**	0.970	0.892 **(< 0.001)**	0.970 **(< 0.001)**
ChatGPT 3.5	0.982	0.964 **(< 0.001)**	0.982 **(< 0.001)**	0.995	0.991 **(< 0.001)**	0.995 **(< 0.001)**	0.958	0.852 **(< 0.001)**	0.958 **(< 0.001)**

**Table 4:** Wilcoxon’s *p* value for the scores given by the two evaluators to the answers provided by the six LLMs on each of two different scorings, 4 weeks apart, and overall Friedman’s *p* value for the scores given by the two evaluators for both dates to the answers provided by the six LLMs. Statistically significant values in bold

LLM	Wilcoxon’s test		Friedman’s test
	Score 1	Score 2	
Copilot [Evaluator 1-2]	0.420	0.851	0.539
ChatGPT 4o [Evaluator 1-2]	0.462	0.357	0.356
Gemini Advanced [Evaluator 1-2]	0.667	0.060	0.485
ChatGPT 4 [Evaluator 1-2]	0.336	0.655	0.336
Gemini [Evaluator 1-2]	0.977	0.417	0.652
ChatGPT 3.5 [Evaluator 1-2]	0.358	0.498	0.346

As a result, an average score was calculated for each LLM from the score of both evaluators given for both dates, to be used in Friedman’s and Wilcoxon tests. Table 5 presents the descriptive statistics for the average scores of the answers provided by the six LLMs. ChatGPT 4 answers were scored as the best, followed by the answers of Gemini Advanced, ChatGPT 4o, ChatGPT 3.5, Gemini, and Copilot.

**Table 5:** Descriptive statistics for the average scores of the answers provided by the six LLMs

Average score	Copilot	ChatGPT 4o	Gemini advanced	ChatGPT 4	Gemini	Chat GPT3.5
Min	3.56	6.88	7.13	6.88	5.94	6.63
Median	5.66	8.19	7.84	8.03	7.38	7.53
Max	6.38	8.69	9.31	9.13	8.44	8.75
Mean	5.41	8.01	8.06	8.08	7.32	7.61
SEM	0.26	0.20	0.23	0.22	0.24	0.23
SD	0.82	0.62	0.73	0.69	0.74	0.73
CoV	15.2%	7.7%	9.1%	8.5%	10.1%	9.5%

*CoV* coefficient of variance, *Max* maximum, *Min* minimum, *SD* standard deviation, *SEM* standard error of mean

According to Kruskal–Wallis test, statistically significant differences were observed between the average scores of the six LLMs (*p* value < 0.001). More specifically, a statistically significant difference was noted between the average scores for Copilot with all other LLMs (*p* value = 0.005 in all the tests), ChatGPT 4o and Gemini (*p* value = 0.014), Gemini Advanced and Gemini (*p* value = 0.019), and ChatGPT 4 and Gemini (*p* value = 0.008) (Table 6). Based on the aforementioned, ChatGPT 4 answers (average score = 8.08), the Gemini Advanced (average score = 8.06), the ChatGPT 4o (average score = 8.01), and the ChatGPT 3.5 (average score = 7.61) scored the best, followed by the Gemini (average score = 7.32) and finally Copilot (average score = 5.41).

**Table 6:** Wilcoxon’s tests *p* value for the average scores of the answers provided by the six LLMs. Statistically significant values in bold

LLM [average scores]	Wilcoxon’s test
Copilot vs. ChatGPT 4o	**0.005**
Copilot vs. Gemini Advanced	**0.005**
Copilot vs. ChatGPT 4	**0.005**
Copilot vs. Gemini	**0.005**
Copilot vs. LLM ChatGPT 3.5	**0.005**
ChatGPT 4o vs. Gemini Advanced	0.722
ChatGPT 4o vs. ChatGPT 4	0.646
ChatGPT 4o vs. Gemini	**0.014**
ChatGPT 4o vs. ChatGPT 35	0.414
Gemini Advanced vs. ChatGPT 4	0.878
Gemini Advanced vs. Gemini	**0.019**
Gemini Advanced vs. ChatGPT 3.5	0.314
ChatGPT 4 vs. Gemini	**0.008**
ChatGPT 4 vs. ChatGPT 3.5	0.153
Gemini vs. ChatGPT 3.5	0.444

**Fig. 1:**
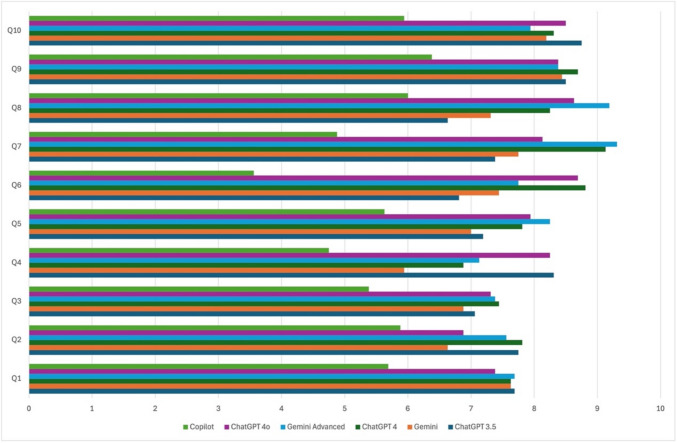
The average scores for the answers to each question provided by the six LLMs

Finally, Fig. 1 presents the average scores of the answers to each question provided by the six LLMs.

## Discussion

AI technology has the potential to make great impacts on dental practice. Examining the accuracy and integrity of AI chatbots is imperative to ensure their role in dental practice. The reliability, validity, and accuracy of chatbots have not been meticulously assessed, particularly when it comes to open-ended questions (Goodman 2023). With the aim to specify the current state of AI in dentistry such as potential benefits, weaknesses or challenges and to predict its possible future progress and application in dentistry, an assessing of six language model chatbots took place.

In the present study, representative questions regarding different paediatric dentistry topics and clinical practice situations were asked to the chatbots. Variations on the results were noted. Chat GPT 4 responses received the highest scores (average score 8.08), followed by Gemini Advanced (average score 8.06), ChatGPT 4o (average score 8.01), ChatGPT 3.5 (average score 7.61), Gemini (average score 7,32), and Copilot (average score 5.41). Statistical analysis revealed that Chat GPT 4 outperformed all other LLMs and the difference was statistically significant.

This observed diversity of ratings among different chatbots is possibly an outcome of their different algorithms, training data, users’ feedback and updates, resulting in variable accuracy, integrity, and different approach to set questions. For example, ChatGPT utilizes advanced NLP (natural language processing) as well as deep learning, capable of producing humanlike answers and is trained on vast corpus data text. On the other hand, Google Gemini core technology uses advanced AI (latest Ultra 1.0 model), making it appropriate to handle complicated tasks such as coding or logical reasoning and creative problem-solving and is possibly trained on large scale, variable dataset, including data relating to medical care. Microsoft Copilot core technology integrates extensive language models, and aims to increase its productivity across Microsoft 365 applications, trained on different datasets. (Warnat-Herresthal 2022). In the study of Giannakopoulos et al. (2023), comparatively evaluating 4 LLMs in clinical questions in the field of dentistry, ChatGPT-4 also received higher rates according to the evaluators. ChatGPT-4’s high score could possibly be related to its large and diverse text datasets training, which allows it to generate consistent, established, and apprehensive responses to certain queries or statements (Sabzalieva 2023).

Despite variations in the scope and detail of responses to the same queries, remarkable similarities were observed. Moreover, with the exception of Copilot, all chatbots managed to achieve a score level above 6.5 on all queries (Fig. 1), exhibiting fairly high scores. Overall, the evaluators encountered accurate and comprehensive responses. Nevertheless, in certain cases, responses were lacking information in comparison to traditional sources of evidence, not reaching the knowledge that is regarded as “standard”. More detailed questions achieved better scores compared to those framed more generally or requiring critical thinking. A possible explanation could be that the performance of language models (LLMs) tends to display sensitivity regarding the details in a set question. Οn this basis, questions that were not asked in sufficient detail may not have been clear enough for the LLMs to elicit a highly acceptable response (Mago 2023, Safi 2020).

This study may demonstrate the potential of AI systems to produce answers to open- ended questions regarding paediatric dentistry. However, their reliability is yet to be fully founded. Considering that, occasionally, LLMs could provide partially incorrect or incomplete answers, the medical, as well as the dental field, cannot regard them as completely trustworthy. Specialized scientific knowledge and access to evidence-based information are of outmost importance (Liu 2023). A question arises as to whether AI machines could suggest correct diagnosis or treatment in complex clinical case scenarios where many factors need to be considered and critical thinking is required. In the study of Mago and Sharma (2023), ChatGPT-3 efficiently responded to 80 queries related to maxillofacial radiology and could therefore serve as an additional source of information for an oral radiologist Nevertheless, it was highlighted that it could not itself stand for in as the primary source (Mago 2023). The study of Goodman et al. (2023) evaluated the accuracy and comprehensiveness of two LLMs (GPT-3.5 and GPT-4) responses to 284 medical queries developed by physicians across 17 specialities. Both LLMs demonstrated high scores across different specialities; however, they were also found unable to grade the reliability of their initial source. In the study of Makrygiannakis et al. (2024), a comparison of the answers given by four LLMs (Google’s Bard, OpenAI’s ChatGPT-3.5, and ChatGPT-4 and Microsoft’s Bing), took place in response to ten clinical questions related to the field of orthodontics. All LLMs occasionally produced answers of low comprehensiveness, accuracy, clarity, and relevance, suggesting that they cannot replace clinical thinking and overall knowledge regardless of their demonstrated ability in supporting evidence-based orthodontics. It should be noted that this study was based on LLMs versions available 1 year ago.

There are several challenges to overcome the inaccuracies of chatbot-provided medical information. Frequently, the LLMs are unable to reach the reliability of the sources of its training data, lacking the relation with established guidelines or evidence-based articles (Goodman 2023). Training LLMs with specific, dentistry-related data, dental terms, and patient records is suggested to ensure their further application in dentistry (Giannakopoulos 2023). Nevertheless, supervised training itself raises the concern of misleading the LLM model depending on individual biases or incomplete knowledge (Goodman 2023). An additional challenge in developing clinical AI systems is to warrant that they are regularly assessed for ensure their accuracy, sensitivity, and specificity over time (Gedefaw 2023).

Although rapid evolution of AI and machine learning could result to advanced chatbots and increase their abilities, their potential to totally substitute medical or dental professionals remains remote as clinical decisions ultimately rely on human judgement. A collaboration between Al chatbots and medical or dental professionals, where LLMs have a supportive and not the primary role, seems, currently, the ideal approach (Carrillo-Perez 2022). LLMs have the potential to assist paediatric dental practitioners in several ways, such as providing instant access to evidence-based treatment guidelines, generating child-friendly educational materials, and synthesizing complex clinical data for caregiver discussions. These capabilities could enhance the efficiency and effectiveness of paediatric dental care while maintaining a patient-centred approach.

Al chatbots have shown potential in supporting clinicians towards efficient and patient-centred care (Mertens 2021). In the present study, ten queries regarding paediatric dentistry were selected, based on clinical relevance and evidence-based paediatric dentistry. However, these questions were indicative and could not cover the whole field of the specialty. Although the responses achieved mostly high scores, limitations and incompleteness were also recorded suggesting that, for the time being, LLMs should not be considered as fully reliable. Al models cannot substitute professionals’ knowledge and critical thinking.

A possible limitation of the present study could be the lack of follow-up questions, as the questions were asked only once. Repeating the questions or requesting clarifications could have possibly led to more accurate answers. As a result, to some extent, some responses might have been underestimated. On the other hand, follow-up questions were not included to avoid bias and unnecessary complications in the research design. Furthermore, this single interaction led to a more focused assessment of the chatbot’s answers in a precise and efficient process, similar to the demands of clinical practice. The concept of a “criterion standard” could also be regarded as a limitation. Clinical recommendations and guidelines may vary worldwide and can change over time. Furthermore, the study evaluated LLM responses during the research period, though their performance could differ over time due to rapid technological evolution.

Extensive research and further clinical validation are essential to consolidate these results. LLMs ability to reply wider or more complex questions or clinical scenarios could also be explored. Moreover, AI model improvements are of outmost importance with the aim to address their limitations. Dental professionals should critically use them as a supportive tool and not as a substitute of their overall knowledge and critical thinking.

## Conclusions

This study demonstrates the potential use of artificial intelligence systems to produce answers to open-ended questions regarding paediatric dentistry. However, their reliability remains still under question. Considering that, occasionally, large language models could provide partially incorrect or incomplete answers, the dental field cannot regard them as completely trustworthy, as they lack the ability of the paediatric dentist’s critical thinking and scientific approach. To ensure the best patient care, paediatric dental professionals should critically use artificial intelligence models as supportive tools and not as a substitute of their overall knowledge and critical thinking. To integrate large language models effectively, dental professionals can use them for tasks such as summarizing guidelines, aiding treatment planning, and generating patient education materials, while verifying accuracy. Artificial intelligence model enhancements and improvements are of utmost importance with the aim to address their limitations. Future research should prioritize refining algorithms to reduce errors, expanding training datasets with paediatric dental literature, and evaluating their clinical applications to ensure their utility in practice.

## Supplementary Information

Below is the link to the electronic supplementary material.

Supplementary file1 (DOCX 134 KB)

Supplementary file2 (DOCX 17 KB)

## Data Availability

The dataset is very extensive and not directly relevant to the reader. Therefore, we have decided not to make it publicly available. However, it can be provided upon reasonable request from the corresponding author.
