# Supplementary material for: Evaluating the evidence-based potential of six large language models in paediatric dentistry: a comparative study on generative artificial intelligence
Source: Eur Arch Paediatr Dent. 2025 Feb 22;26(3):527–35. doi: 10.1007/s40368-025-01012-x (PMC12165978; doi:10.1007/s40368-025-01012-x)
Supplement: Supplementary file 1 — Supplementary file1 (DOCX 134 KB) [file 40368_2025_1012_MOESM1_ESM.docx]

**Supplementary Table 1**

| **Questions**  **discipline** | **ChatGPT 3.5** | **ChatGPT 4** | **Chat GPT 4o** [**‎**](https://bard.google.com/) | **GEMINI** | **GEMINI ADNANCED** | **COPILOT** | **Evidence (literature)** |
| --- | --- | --- | --- | --- | --- | --- | --- |
| **1.** When and how should a dentist use local analgesia to control pain in children, adolescents, and medically compromised individuals during the delivery of oral health care? | Local analgesia, commonly referred to as local anesthesia, is a crucial tool in dentistry for managing pain during various procedures. Its usage in children, adolescents, and medically compromised individuals requires careful consideration and tailored approaches due to differences in anatomy, physiology, and medical conditions. Here's a general guide on when and how dentists should use local analgesia for these groups:   1. **Children:**    - **When to Use:** Local analgesia is often necessary for invasive dental procedures or treatments that may cause discomfort or pain.    - **Considerations:** Children may have heightened anxiety or fear associated with dental procedures, which can affect pain perception. Child-friendly language, behavior management techniques, and distractions can help alleviate anxiety and enhance the effectiveness of local analgesia.    - **Technique:** Pediatric dentists often use techniques such as tell-show-do, topical anesthetics, and distraction techniques to minimize discomfort during administration. Child-sized needles and appropriate injection sites are used to minimize pain and trauma. 2. **Adolescents:**    - **When to Use:** Similar to adults, local analgesia is commonly used for adolescents during dental procedures such as fillings, extractions, or root canal treatments.    - **Considerations:** Adolescents may have a higher pain threshold compared to younger children but may still experience anxiety or fear related to dental procedures. Communication about the procedure and pain management options is important.    - **Technique:** Standard local anesthesia techniques applicable to adults can be used for adolescents. Dentists may opt for topical anesthetics and distraction techniques to enhance patient comfort during administration. 3. **Medically Compromised Individuals:**    - **When to Use:** Local analgesia is essential for medically compromised individuals to ensure they can undergo necessary dental treatments comfortably.    - **Considerations:** Medical conditions and medications may affect the choice of anesthetic agents, dosage, and administration techniques. Close collaboration with the patient’s physician or specialist may be necessary to ensure safe and effective pain management.    - **Technique:** Dentists may need to adjust the dosage and type of local anesthetic based on the patient's medical condition and any medications they are taking. Careful monitoring during and after administration is important to detect any adverse reactions or complications.   In all cases, dentists should prioritize patient comfort and safety when using local analgesia. Proper assessment of the patient's medical history, anxiety levels, and procedural needs, along with effective communication, can help optimize pain management during dental treatments. Additionally, ongoing education and training in pediatric dentistry, adolescent dentistry, and managing medically compromised patients can further enhance a dentist's ability to provide effective pain control. | Here are some guidelines on when and how a dentist should use local anesthesia:  **1. Assessment:**   - Perform a thorough medical history evaluation to identify any contraindications or precautions. - Determine the level of anxiety and the pain threshold of the patient. - Consider the nature and extent of the dental procedure.   **2. Technique:**   - Use a topical anesthetic to numb the area before injecting the local anesthetic to minimize injection discomfort. - Employ the slow injection technique, as it is less painful and allows for better diffusion of the anesthetic. - Use distraction techniques for children to reduce their focus on the injection.   **3. Choice of Anesthetic:**   - For shorter procedures, a dentist might use anesthetics with a shorter duration of action to minimize the period of numbness after the procedure. - For longer procedures or those that might cause significant postoperative pain, a longer-acting anesthetic may be chosen.   **4. Dosage:**   - Calculate the appropriate dose based on the patient's weight, especially for children, to avoid toxicity. - For medically compromised individuals, adjust the dosage and type of anesthetic according to their medical condition and medications.   **5. Monitoring:**   - Monitor the patient for signs of adverse reactions during and after the administration of local anesthesia. - Ensure that proper emergency equipment and medications are readily available in case of an anaphylactic reaction or other emergencies.   **6. Postoperative Care:**   - Advise patients and their caregivers about the care of numb areas, particularly to avoid biting or injuring the soft tissues. - Follow up on any adverse reactions or prolonged numbness.   **7. Documentation:**   - Record the type, amount, and site of anesthesia administered. - Note any reactions or complications during the follow-up.   **8. Education and Communication:**   - Explain the procedure to the patient and their caregivers, especially when treating children, to gain their cooperation. - Reassure them that local anesthesia is a safe and effective way to prevent pain during dental treatments.   **9. Consent:**   - Obtain informed consent after discussing the benefits and risks of local anesthesia with the patient or their guardians.   Each patient is unique, and the approach should be tailored to the individual's needs, considering their medical, psychological, and dental status. In case of specific medical conditions, consulting the patient's physician may be necessary to ensure safe management. | Based on the provided image, the document seems to be addressing guidelines for the use of local anesthesia in different patient groups including children, adolescents, and medically compromised individuals during dental procedures.  **Local Anesthesia in Pediatric Patients:**   1. **Age-Appropriate Techniques**: Use techniques appropriate for the child’s age and developmental level. 2. **Dose Adjustment**: Adjust the dose of local anesthetic based on the child’s weight to avoid overdose. 3. **Behavior Management**: Employ behavior management techniques to alleviate anxiety and ensure cooperation. 4. **Topical Anesthetic**: Apply topical anesthetic before injection to reduce the pain of needle insertion. 5. **Informed Consent**: Obtain informed consent from the parents or guardians, explaining the procedure and its benefits.   **Local Anesthesia in Adolescents:**   1. **Individual Assessment**: Consider the maturity level and medical history of the adolescent. 2. **Effective Communication**: Clearly explain the procedure and address any fears or concerns. 3. **Appropriate Dosage**: Calculate the dose based on the adolescent’s weight and medical history. 4. **Pain Management**: Use a combination of topical and local anesthetics to ensure effective pain control. 5. **Monitoring**: Continuously monitor the patient for any adverse reactions during and after the procedure.   **Local Anesthesia in Medically Compromised Individuals:**   1. **Medical History Review**: Thoroughly review the patient’s medical history and current medications. 2. **Consultation**: Consult with the patient’s physician if necessary to understand any medical conditions that might affect the use of local anesthesia. 3. **Modified Techniques**: Modify anesthetic techniques and dosages based on the individual’s medical condition. 4. **Emergency Preparedness**: Be prepared for medical emergencies by having emergency equipment and medications readily available. 5. **Close Monitoring**: Closely monitor the patient’s vital signs during the procedure and be alert for any signs of complications.   **General Guidelines for All Patient Groups:**   1. **Selection of Anesthetic**: Choose an appropriate local anesthetic based on the duration of the procedure and the patient’s medical history. 2. **Sterility**: Ensure all equipment is sterile to prevent infections. 3. **Injection Technique**: Use proper injection techniques to minimize pain and ensure effective anesthesia. 4. **Post-Operative Instructions**: Provide clear post-operative instructions to manage pain and prevent complications. 5. **Documentation**: Document the type, dosage, and administration site of the local anesthetic in the patient’s medical record.   These guidelines are aimed at ensuring safe and effective pain management during dental procedures for children, adolescents, and medically compromised individuals. Always tailor the approach to the specific needs of each patient. | Local analgesia is a valuable tool for dentists to manage pain in children, adolescents, and medically compromised individuals. Here's a breakdown of when and how it should be used:  **When to Use Local Analgesia:**   - **Painful Procedures:** Local anesthesia is generally recommended for any procedure that is likely to cause discomfort, such as fillings, extractions, or root canals. - **Anxiety and Fear:** Even non-invasive procedures can be anxiety-provoking for some patients. Local anesthesia can help reduce anxiety by ensuring they won't feel pain. - **Behavioral Management:** For children or patients with cognitive difficulties who may struggle to cooperate during treatment, local anesthesia can help ensure a smoother and safer procedure. - **Medically Compromised Individuals:** Some medical conditions can make patients more sensitive to pain. Local anesthesia can minimize discomfort and ensure they can tolerate the procedure.   **How to Use Local Analgesia:**   - **Choosing the Right Agent:** The dentist will select the most appropriate local anesthetic based on factors like the type of procedure, patient age, and medical history. - **Minimizing Discomfort:** Techniques like topical anesthetics (numbing gels) applied before injection, anesthetic warming, and gentle injection methods can help reduce the discomfort associated with the injection itself. - **Dosage Considerations:** The dentist will carefully determine the appropriate dosage of local anesthetic for the specific patient and procedure, considering factors like age and weight. - **Communication and Age-Appropriate Techniques:** For children and adolescents, dentists will use clear and age-appropriate communication to explain the procedure and address any anxieties. Techniques like distraction and positive reinforcement can also be helpful. - **Collaboration with Medical Professionals:** For medically compromised individuals, the dentist may consult with the patient's physician to ensure the chosen local anesthetic and dosage are safe for their specific condition.   Here are some resources for further reading:   - American Academy of Pediatric Dentistry: <https://www.aapd.org/research/oral-health-policies--recommendations/use-of-local-anesthesia-for-pediatric-dental-patients/> - European Academy of Paediatric Dentistry: <https://pubmed.ncbi.nlm.nih.gov/28913645/>   Remember, this information is intended for general knowledge only. It's always best to consult with a qualified dentist to discuss the best pain management options for your specific needs | The use of local analgesia in children, adolescents, and medically compromised individuals requires careful consideration and adherence to best practices to ensure both effective pain control and safety.  **When to Use Local Analgesia:**  Local analgesia should be used whenever a dental procedure is likely to cause pain or discomfort. This includes procedures like:   - **Cavity preparation and filling** - **Tooth extractions** - **Root canals** - **Scaling and root planing (deep cleaning)** - **Injections**   The decision to use local analgesia should be based on a thorough assessment of the patient's individual needs, medical history, and the nature of the planned procedure.  **How to Use Local Analgesia:**   1. **Topical Anesthesia:** Before administering an injection, topical anesthesia should be applied to numb the injection site. This can significantly reduce the discomfort associated with the injection. 2. **Injection Technique:** The injection should be given slowly and steadily, with constant communication with the patient to gauge their comfort level. Aspiration (pulling back on the syringe to check for blood) should be performed before injecting to avoid injecting into a blood vessel. 3. **Dosage:** The appropriate dosage of local anesthetic should be calculated based on the patient's weight and age, and the type of procedure being performed. 4. **Monitoring:** The patient should be monitored closely during and after the procedure for any signs of adverse reactions or complications.   **Special Considerations:**   - **Children:** Children may require additional behavioral management techniques to ensure their cooperation during the administration of local anesthesia. - **Adolescents:** Adolescents may have concerns about the safety and potential side effects of local anesthesia, so it is important to address their questions and provide reassurance. - **Medically Compromised Individuals:** It is crucial to consult with the patient's physician or specialist to determine the most appropriate type and dosage of local anesthetic, taking into account any underlying medical conditions or medications.   **Important Note:** The use of local analgesia in dentistry is generally safe and effective when administered by a qualified professional following established guidelines and best practices.  For more detailed information, you may refer to the following resources:   - Best clinical practice guidance for local analgesia in paediatric dentistry: an EAPD policy document: <https://pubmed.ncbi.nlm.nih.gov/29022286/> - A systematic review of the use of local analgesia in medically compromised children and adolescents: <https://pubmed.ncbi.nlm.nih.gov/28983877/>   Please remember to consult with your dentist or orthodontist for personalized advice and treatment recommendations. | **Local anesthesia** plays a crucial role in **managing pain** during dental procedures for children, adolescents, and medically compromised individuals. Here are some key points:   1. **Appropriate Use**: Dentists should judiciously use local analgesia to control pain during dental treatments. This involves selecting the right anesthetic agent and dosage, as well as choosing the proper route of administration. 2. **Clinical Effectiveness**: When administered correctly, local analgesia is **clinically effective** in treating children. It helps control pain and minimizes discomfort during dental procedures. 3. **Low Risk**: Local analgesia carries a **very low risk** of adverse effects or side effects when used appropriately. Dentists can confidently use it to enhance patient comfort. 4. **Knowledge Gaps**: Despite its benefits, there are gaps in knowledge regarding local analgesia. For instance, in several European countries, the commonly used injectable local anesthetic agent, articaine, is not approved for children under the age of 4 years. 5. [**Research Needs**: To improve pain management for vulnerable patients, further research is needed to address these gaps in knowledge^1^](https://link.springer.com/article/10.1007/s40368-017-0311-y). 6. [Remember that pain control is essential for building trust, reducing anxiety, and promoting positive dental experiences for young patients^2^](https://iapdworld.org/wp-content/uploads/2022/08/2022_15_local-anaesthesia-in-pediatric-dentistry.pdf). [Dentists should stay informed about best practices and adapt their approach based on individual patient needs^3^](https://www.aapd.org/media/Policies_Guidelines/BP_Pain.pdf). 😊 | Kühnisch J, Daubländer M, Klingberg G, Dougall A, Spyridonos Loizides M, Stratigaki E, Amar JL, Anttonen V, Duggal M, Gizani S. Best clinical practice guidance for local analgesia in paediatric dentistry: an EAPD policy document. Eur Arch Paediatr Dent. 2017 Oct;18(5):313-321. doi: 10.1007/s40368-017-0311-y. PMID: 29022286 |
|  | **ChatGPT 3.5** | **ChatGPT 4** | **Chat GPT 4o** [**‎**](https://bard.google.com/) | **GEMINI** | **GEMINI ADNANCED** | **COPILOT** | **Evidence (literature)** |
| **2.** What is the etiology of molar-incisor-hypomineralization (MIH)? What are the best clinical practices for preventive and treatment options for MIH teeth according to their severity? | Molar-Incisor Hypomineralization (MIH) is a developmental dental condition characterized by enamel defects affecting one or more first permanent molars and often incisors. The exact etiology of MIH is not fully understood, but it's believed to be multifactorial, involving genetic, environmental, and systemic factors. Some potential factors contributing to MIH include:   1. **Genetic Factors:** There is evidence to suggest a genetic predisposition to MIH, with certain gene polymorphisms being associated with the condition. However, the specific genetic mechanisms involved are still being investigated. 2. **Environmental Factors:** Prenatal and perinatal factors, such as maternal illness during pregnancy, premature birth, low birth weight, and early childhood illnesses, have been implicated in the development of MIH. Environmental factors during tooth development, such as exposure to environmental toxins or disruptions in mineral metabolism, may also play a role. 3. **Systemic Factors:** Systemic conditions or disturbances during the mineralization of permanent teeth, such as fever episodes or childhood illnesses, may affect enamel formation and contribute to MIH.   Clinical management of MIH involves both preventive measures and treatment options, which vary depending on the severity of the condition:  **Preventive Measures:**   1. **Early Detection:** Regular dental examinations, including visual inspection and palpation, can facilitate early detection of MIH lesions. Early intervention allows for timely preventive measures and treatment planning. 2. **Oral Hygiene:** Emphasize good oral hygiene practices, including regular brushing with fluoride toothpaste and flossing, to maintain oral health and prevent further deterioration of affected teeth. 3. **Dietary Counseling:** Educate patients and caregivers about the importance of a balanced diet low in sugary and acidic foods and beverages to minimize the risk of enamel demineralization and caries. 4. **Fluoride Therapy:** Topical fluoride applications, such as professionally applied fluoride varnishes or fluoride-containing dental products, can help strengthen enamel and reduce the risk of caries development in MIH-affected teeth.   **Treatment Options:**   1. **Microabrasion:** Mild cases of MIH may benefit from microabrasion techniques to remove superficial enamel defects and improve aesthetics. 2. **Resin Infiltration:** Resin infiltration involves the application of low-viscosity resin into the porous enamel of MIH-affected teeth to improve esthetics and reduce sensitivity. 3. **Composite Restorations:** Composite resin restorations can be used to restore aesthetics and function in MIH-affected teeth with moderate enamel defects or cavitations. 4. **Stainless Steel Crowns:** In cases of severe enamel defects or extensive caries involvement, stainless steel crowns may be indicated to provide long-term protection and preservation of affected teeth. 5. **Extraction and Orthodontic Treatment:** In cases of severe structural defects or extensive caries leading to tooth loss, extraction followed by orthodontic treatment may be necessary to maintain proper occlusion and dental arch integrity.   Treatment planning for MIH should be individualized based on the severity of enamel defects, presence of symptoms (such as sensitivity or pain), patient age, and aesthetic considerations. Close collaboration between the dentist, patient, and caregivers is essential to develop a comprehensive management plan aimed at preserving dental health and function while addressing the esthetic concerns associated with MIH. | Molar-Incisor Hypomineralization (MIH) is a developmental condition characterized by the hypomineralization of systemic origin of one to four first permanent molars, frequently affecting the permanent incisors as well. Here is an overview of the etiology, prevention, and treatment of MIH based on its severity:  **Etiology of MIH**  The exact cause of MIH is not completely understood, but several factors are believed to contribute to its development:   1. **Genetic Factors**: There might be a hereditary component influencing susceptibility to MIH. 2. **Environmental Factors**: Prenatal and perinatal issues such as maternal illness, nutritional deficiencies, or premature birth are potential risk factors. 3. **Childhood Illnesses**: Illnesses during the first three years of life, particularly those involving high fevers, are commonly associated with the development of MIH. 4. **Exposure to Dioxins**: Some studies suggest that exposure to environmental pollutants like dioxins during early childhood may play a role.   **Preventive and Treatment Options for MIH**  Preventive and treatment strategies for MIH vary based on the severity of the condition:  **Mild MIH**   - **Prevention**:   - Regular dental check-ups to monitor the progression.   - Application of fluoride varnish to enhance enamel hardness and resistance.   - Good oral hygiene practices to prevent caries. - **Treatment**:   - Microabrasion to remove superficial discoloration.   - Dental composite restorations to cover aesthetic defects and protect the enamel.   **Moderate MIH**   - **Prevention**:   - Use of fluoride supplements if the drinking water is not fluoridated.   - Protective sealants on molars to prevent decay. - **Treatment**:   - Resin infiltration technique to mask white spots and prevent progression of enamel breakdown.   - In cases where the enamel is structurally compromised, indirect restorations like inlays or onlays can be considered.   **Severe MIH**   - **Prevention**:   - Early intervention is crucial to manage severe cases; this might include more frequent dental visits and possibly diet modifications to support enamel health. - **Treatment**:   - Full coverage crowns to restore function and aesthetics, especially if the molars are structurally weakened.   - Extraction may be necessary in cases where the tooth is non-restorable, followed by orthodontic treatment to manage the space and prepare for future restorations like dental implants after growth completion.   In all cases, the approach to managing MIH involves a combination of aesthetic considerations and protective measures to maintain dental function and reduce sensitivity. Regular follow-up and customized treatment planning are essential to address this condition effectively, as the needs can vary widely from one patient to another. Additionally, patient and parent education on the condition is critical to ensuring adherence to preventive measures and treatment regimens. | **Etiology of Molar-Incisor Hypomineralization (MIH)**  Molar-incisor hypomineralization (MIH) is a developmental condition affecting the enamel of first permanent molars and often permanent incisors. The etiology of MIH is multifactorial, involving genetic and environmental factors. Key etiological factors include:   1. **Prenatal and Perinatal Influences**:    - Maternal illness or medication during pregnancy    - Premature birth or low birth weight    - Complications during delivery 2. **Childhood Illnesses and Environmental Factors**:    - Frequent episodes of high fever during early childhood    - Respiratory diseases and infections    - Exposure to environmental toxins such as dioxins    - Nutritional deficiencies, particularly in calcium and vitamin D 3. **Genetic Predisposition**:    - Family history of dental anomalies    - Genetic variations influencing enamel formation   **Best Clinical Practices for Preventive and Treatment Options for MIH Teeth According to Their Severity**  **Preventive Measures:**   1. **Regular Dental Check-ups**:    - Early diagnosis through regular dental visits    - Monitoring the progression of hypomineralization 2. **Oral Hygiene Education**:    - Emphasis on proper brushing techniques    - Use of fluoride toothpaste to strengthen enamel 3. **Dietary Advice**:    - Reducing consumption of acidic and sugary foods and drinks    - Encouraging a balanced diet rich in calcium and vitamin D   **Treatment Options Based on Severity:**  **Mild MIH**:   - **Fluoride Treatments**: Application of fluoride varnishes to enhance enamel strength and reduce sensitivity. - **Desensitizing Agents**: Use of products containing potassium nitrate or calcium phosphate to alleviate sensitivity. - **Sealants**: Application of resin-based sealants to protect the occlusal surfaces of molars.   **Moderate MIH**:   - **Restorative Treatments**: Composite resin restorations to repair affected areas and improve aesthetics. - **Microabrasion**: Removal of superficial enamel defects combined with remineralization therapies. - **Infiltration Techniques**: Resin infiltration to mask discolorations and stabilize the enamel.   **Severe MIH**:   - **Full-Coverage Restorations**: Placement of preformed stainless steel crowns on molars to protect the tooth structure and reduce sensitivity. - **Indirect Restorations**: Use of onlays or crowns for extensive enamel loss or severe structural compromise. - **Endodontic Treatment**: In cases of pulp involvement, root canal therapy followed by appropriate restorative treatment.   **Pain and Hypersensitivity Management**:   - **Topical Anesthetics**: Application of topical anesthetic gels or creams before treatment. - **Analgesics**: Use of pain-relieving medications to manage discomfort. - **Behavioral Techniques**: Techniques such as distraction and relaxation to help manage anxiety and pain during treatment.   **Summary**  Managing MIH requires a tailored approach based on the severity of the condition. Early diagnosis and preventive measures can significantly improve outcomes. For mild cases, fluoride treatments, sealants, and desensitizing agents are effective. Moderate cases may require restorative treatments and microabrasion, while severe cases often necessitate full-coverage restorations and endodontic treatment. Effective pain and hypersensitivity management is crucial across all severity levels to ensure patient comfort and cooperation. | **Molar-Incisor-Hypomineralization (MIH): Unveiling the Cause and Course of Action**  Molar-incisor-hypomineralization (MIH) is a dental defect affecting the enamel development of permanent teeth, primarily targeting the first permanent molars and incisors. While the exact cause (etiology) of MIH remains elusive, research suggests several potential factors:   - **Prenatal and Early Childhood Health:** Certain prenatal or early childhood events might play a role, such as maternal infections, prematurity, high fever episodes, or specific childhood illnesses like asthma. - **Environmental Factors:** Exposure to environmental toxins during tooth development is a potential contributing factor, though more research is needed.   **Shining a Light on Preventive and Treatment Options**  Unfortunately, there's no way to reverse existing MIH damage. However, depending on the severity, dentists can recommend different approaches:  **Preventive Measures:**   - **Maintaining Good Oral Hygiene:** Regular brushing and flossing are crucial to prevent dental caries (cavities) that MIH teeth are more susceptible to. - **Fluoride Therapy:** Professional topical fluoride applications or fluoride toothpaste can help strengthen enamel and reduce sensitivity.   **Treatment Options based on MIH Severity:**   - **Mild MIH:** May require minimal intervention beyond preventive measures. - **Moderate MIH:** Depending on the degree of discoloration or enamel defects, treatments like microabrasion (removal of a thin layer of enamel) or tooth bonding (composite resin applied to improve aesthetics) might be recommended. - **Severe MIH:** In severe cases with significant defects or compromised tooth structure, crowns or veneers might be necessary to restore functionality and aesthetics.   **Additional Considerations:**   - **Early Detection:** Regular dental checkups are essential for early detection and prompt intervention to manage MIH effectively. - **Addressing Sensitivity:** MIH teeth can be sensitive to cold or hot stimuli. Desensitizing toothpastes or specific treatments can help alleviate discomfort. - **Psychological Impact:** The cosmetic concerns associated with MIH can affect a child's self-esteem. Addressing these concerns and providing reassurance can be equally important.   Remember, consulting a qualified dentist is crucial to determine the best course of action for managing MIH based on the individual's specific situation and severity of the condition. | Molar-incisor hypomineralization (MIH) is a qualitative enamel defect of systemic origin affecting one or more first permanent molars, often with affected permanent incisors.  **Etiology:**  The exact cause of MIH remains unclear, but it is believed to be multifactorial. Proposed etiological factors include:   - **Prenatal factors:** Maternal illness, medication use during pregnancy, prematurity, and birth complications. - **Early childhood illnesses:** Fever, asthma, pneumonia, otitis media (ear infections), and urinary tract infections during the first 3 years of life. - **Environmental factors:** Exposure to environmental toxins and pollutants. - **Genetic predisposition:** Possible genetic susceptibility in some individuals.   **Best Clinical Practices for Prevention and Treatment:**  Prevention:  Since the etiology of MIH is not fully understood, there are no definitive preventive measures. However, promoting overall maternal and child health during the perinatal and early childhood periods may help reduce the risk of MIH.  Treatment:  The treatment of MIH depends on the severity of the defect and the associated symptoms. Treatment options include:   - **Mild MIH (White or cream opacities):**   - No treatment or preventive resin infiltration for esthetic concerns.   - Topical fluoride application to prevent caries.   - Regular dental checkups to monitor for any changes. - **Moderate MIH (Yellow or brown opacities, enamel breakdown):**   - Microabrasion for esthetic improvement.   - Composite resin restorations to repair enamel defects.   - Glass ionomer cement (GIC) restorations for tooth-colored fillings.   - Preformed metal crowns (PMCs) for extensive breakdown in primary teeth.   - Stainless steel crowns (SSCs) for extensive breakdown in permanent molars. - **Severe MIH (Extensive enamel breakdown, post-eruptive breakdown, dentin sensitivity):**   - Extensive restorative treatment with composite resin or GIC.   - Full coverage crowns for severely affected teeth.   - Endodontic treatment (root canal) if the pulp is involved.   **Additional Considerations:**   - MIH teeth are more susceptible to dental caries (cavities), so meticulous oral hygiene and regular dental visits are crucial. - Dental anxiety and fear are common in children with MIH due to increased sensitivity and the need for dental treatment. Providing a supportive and comfortable environment is essential for successful treatment outcomes.   **Important Note:** It is important to consult with a dentist or pediatric dentist for a comprehensive evaluation and personalized treatment plan for MIH. | **Molar-Incisor Hypomineralization (MIH)** is a developmental enamel defect that affects at least one first permanent molar and occasionally the incisors. Let’s delve into the etiology and management:   1. **Etiology**:    - **Multifactorial**: MIH has a multifactorial etiology, involving systemic, genetic, and environmental factors.    - **Perinatal Factors**: Perinatal hypoxia, prematurity, and related issues (e.g., caesarean section) may increase MIH risk.    - **Childhood Illnesses**: Certain childhood illnesses are linked to MIH.    - **Genetic Predisposition**: Twin studies and genetic analyses suggest a genetic influence.    - [**Epigenetic Factors**: Epigenetic influences play a role^1^](https://www.mdpi.com/2227-9067/8/6/466). 2. **Clinical Management**:    - **Severity-Based Approach**:      - **Mild Cases**: Monitor and provide preventive measures.      - **Moderate Cases**: Consider fluoride varnish, microabrasion, or resin infiltrants for esthetic improvement.      - [**Severe Cases**: Options include composite restorations, preformed metal crowns, or extractions^2^](https://www.eapd.eu/uploads/files/MIH_Best_Practice.pdf)    - **Front Teeth (Incisors)**:      - **Fluoride Varnish**: Reduces sensitivity.      - **Microabrasion**: Improves appearance (may slightly damage teeth).      - **Resin Infiltrant**: Alters light reflection for better aesthetics.      - **Tooth Whitening**: Improves tooth color (use with caution in under 18-year-olds).      - [**White Fillings**: Thin layer over the front of the tooth^3^](https://www.kch.nhs.uk/wp-content/uploads/2023/01/pl-1074.1-molar-incisor-hypomineralisation-mih-in-children.pdf).    - **Research Needs**:      - Investigate systemic etiological factors’ impact on normal amelogenesis.      - Focus on pre-eruption diagnosis and prevention of post-eruption breakdown and caries.      - [Explore new materials and techniques, such as digital dentistry^4^](https://www.mdpi.com/2304-6767/11/7/157).   Remember, early diagnosis and tailored management are crucial for improving the quality of life for individuals with MIH. | Lygidakis NA, Garot E, Somani C, Taylor GD, Rouas P, Wong FSL. Best clinical practice guidance for clinicians dealing with children presenting with molar-incisor-hypomineralisation (MIH): an updated European Academy of Paediatric Dentistry policy document. Eur Arch Paediatr Dent. 2022 Feb;23(1):3-21. doi: 10.1007/s40368-021-00668-5. PMID: 34669177 |
|  | **ChatGPT 3.5** | **ChatGPT 4** | **Chat GPT 4o** [**‎**](https://bard.google.com/) | **GEMINI** | **GEMINI ADNANCED** | **COPILOT** | **Evidence (literature)** |
| **3.** In which clinical situations in pediatric dentistry might detailed imaging with periapical and bitewing radiographs be beneficial? | Periapical and bitewing radiographs are valuable diagnostic tools in pediatric dentistry, providing detailed imaging of the teeth and surrounding structures. These imaging techniques are beneficial in various clinical situations for both diagnostic and treatment planning purposes. Here are some scenarios in pediatric dentistry where periapical and bitewing radiographs may be beneficial:   1. **Assessment of Dental Caries:**    - Periapical and bitewing radiographs enable the detection of dental caries in primary and permanent teeth, particularly in areas not visible during clinical examination, such as interproximal surfaces and areas beneath existing restorations. 2. **Evaluation of Dental Development:**    - Radiographs help assess the developmental stage of primary and permanent teeth, including eruption patterns, root development, and presence of anomalies such as supernumerary or congenitally missing teeth. 3. **Detection of Dental Trauma:**    - Periapical radiographs are useful for assessing traumatic injuries to primary and permanent teeth, including fractures, luxations, and avulsions. They help determine the extent of dental trauma and guide appropriate treatment planning. 4. **Assessment of Periodontal Health:**    - Periapical radiographs provide information about the health of the supporting structures of the teeth, including the alveolar bone and periodontal ligament. They aid in the diagnosis of periodontal disease, bone loss, and abnormalities in the periodontal ligament space. 5. **Identification of Pathologies:**    - Radiographs help detect various pathological conditions affecting the teeth and jaws, such as dental abscesses, cysts, tumors, and developmental anomalies. Early detection of these pathologies is crucial for timely intervention and prevention of complications. 6. **Evaluation of Orthodontic Needs:**    - Radiographs are essential for assessing the alignment, eruption patterns, and skeletal relationships of the teeth and jaws in pediatric patients requiring orthodontic treatment. They help orthodontists develop comprehensive treatment plans and monitor treatment progress. 7. **Monitoring of Dental Treatment:**    - Periapical and bitewing radiographs are used to monitor the effectiveness of dental treatments, such as restorations, pulp therapy, and orthodontic interventions. They provide visual evidence of treatment outcomes and help identify any complications or recurrence of dental problems. 8. **Assessment of Dental Anatomy and Morphology:**    - Radiographs assist in evaluating the anatomy and morphology of primary and permanent teeth, including the presence of developmental anomalies, variations in tooth morphology, and abnormalities in tooth structure.   In summary, periapical and bitewing radiographs play a crucial role in pediatric dentistry by providing detailed imaging of the teeth and supporting structures. They aid in the diagnosis, treatment planning, and monitoring of various dental conditions, thereby contributing to the delivery of comprehensive and quality dental care for pediatric patients. | Detailed imaging with periapical and bitewing radiographs plays a crucial role in pediatric dentistry for diagnostic and treatment planning purposes. These types of radiographs provide detailed views of the teeth and supporting structures, which are essential in several clinical situations:   1. **Caries Detection**:    - **Bitewing Radiographs**: These are particularly useful for detecting interproximal decay, which might not be visible clinically. Bitewings are crucial for assessing the extent of caries and for monitoring their progression over time in the posterior teeth.    - **Periapical Radiographs**: These help in detecting caries that are close to the pulp or have affected the root structure. 2. **Developmental Anomalies**:    - **Periapical Radiographs**: Useful for assessing anomalies in tooth development, eruption patterns, and the presence of supernumerary teeth or congenitally missing teeth.    - Detailed imaging helps in planning orthodontic treatment and in managing impacted teeth. 3. **Trauma Assessment**:    - **Periapical Radiographs**: Essential for evaluating the extent of injury to the dental structures and supporting bone, especially in cases of tooth displacement or avulsion.    - They help in diagnosing root fractures and the status of the developing apex in children. 4. **Assessment of Pulpal and Periapical Status**:    - **Periapical Radiographs**: These are critical for evaluating diseases of the pulp and periapical tissues. They provide views of the entire length of the tooth, including the root and surrounding bone, which is necessary for diagnosing abscesses, cysts, and granulomas. 5. **Orthodontic Assessment**:    - **Periapical and Bitewing Radiographs**: While panoramic radiographs are generally preferred for overall orthodontic assessments, periapical and bitewing radiographs can provide detailed information about specific areas, such as the relation of the roots to each other and to the cortical bone, which is vital for planning tooth movement. 6. **Periodontal Disease**:    - **Bitewing Radiographs**: They reveal the level of bone support around the teeth, detect bone loss, and help in the assessment of periodontal disease severity. 7. **Monitoring of Dental Development and Eruption**:    - **Periapical Radiographs**: Useful in monitoring the development and eruption of primary and permanent teeth, especially in cases where there are delays or abnormalities in eruption patterns.   These imaging techniques are integral tools in pediatric dentistry, aiding significantly in providing high-quality care tailored to the specific needs of children. Their appropriate use, combined with clinical examination, allows for comprehensive assessment and management of various dental conditions in pediatric patients. | **Clinical Situations in Pediatric Dentistry for Detailed Imaging with Periapical and Bitewing Radiographs**  Detailed imaging with periapical and bitewing radiographs is beneficial in several clinical situations in pediatric dentistry. These radiographic techniques provide critical information for accurate diagnosis and effective treatment planning. Here are the key clinical situations where these radiographs are particularly useful:  **1. Detection of Caries:**   - **Bitewing Radiographs**: Essential for detecting interproximal caries in primary and permanent teeth, particularly in areas not visible during a clinical examination. - **Periapical Radiographs**: Useful for identifying caries that extend into the pulp or involve the root structures.   **2. Assessment of Pulpal and Periapical Pathologies:**   - **Periapical Radiographs**: Provide detailed images of the entire tooth, including the root and surrounding bone, which is critical for diagnosing pulpal infections, periapical abscesses, and other endodontic conditions.   **3. Evaluation of Trauma:**   - **Periapical Radiographs**: Necessary for assessing the extent of dental trauma, such as root fractures, luxations, and avulsions. They help determine the position and condition of the tooth and surrounding bone. - **Bitewing Radiographs**: Can be used to evaluate the integrity of the crown and detect any fractures or injuries.   **4. Monitoring Dental Development:**   - **Bitewing Radiographs**: Useful for monitoring the eruption patterns and development of primary and permanent teeth, ensuring proper spacing and alignment. - **Periapical Radiographs**: Help assess the development and positioning of unerupted or impacted teeth, such as permanent molars and canines.   **5. Periodontal Health Evaluation:**   - **Bitewing Radiographs**: Provide information on the height and health of the alveolar bone and can detect early signs of periodontal disease. - **Periapical Radiographs**: Allow for a more detailed examination of the periodontal structures, including the detection of bone loss and assessment of furcation involvement.   **6. Assessment of Developmental Anomalies:**   - **Periapical Radiographs**: Crucial for diagnosing developmental anomalies such as supernumerary teeth, congenitally missing teeth, and enamel or dentin defects. - **Bitewing Radiographs**: Can aid in identifying anomalies affecting the crowns of the teeth.   **7. Evaluation of Restorative Treatments:**   - **Bitewing Radiographs**: Used to assess the fit and integrity of restorations, such as fillings and crowns, ensuring there are no gaps or recurrent caries. - **Periapical Radiographs**: Useful for evaluating the success of endodontic treatments and ensuring the restoration does not affect the root or periapical area.   **8. Pre-Orthodontic Assessment:**   - **Bitewing and Periapical Radiographs**: Both types of radiographs can be part of the comprehensive orthodontic assessment, providing information on the position and health of the teeth and supporting structures.   **Conclusion**  Detailed imaging with periapical and bitewing radiographs plays a vital role in pediatric dentistry. These radiographic techniques provide essential diagnostic information that aids in the detection of caries, assessment of trauma, monitoring of dental development, evaluation of periodontal health, and more. Proper use of these imaging modalities ensures comprehensive care and effective treatment planning for pediatric patients. | In pediatric dentistry, detailed imaging with periapical and bitewing radiographs proves beneficial in various clinical situations. Here's a breakdown of some key scenarios:  **Diagnosing Caries:**   - **Extensive Caries:** When a cavity is large and extends towards the pulp (inner tooth tissue), a periapical radiograph provides a clearer picture of the damage and helps determine if root canal treatment is necessary. - **Proximal Caries (between teeth):** Bitewing radiographs excel at revealing decay between teeth, especially in areas difficult to examine visually or with probing.   **Evaluating Tooth Development and Eruption:**   - **Monitoring Tooth Bud Development:** Periapical radiographs can track the development and timing of permanent tooth buds beneath the gums, especially if there are concerns about delayed eruption. - **Impacted Teeth:** When a tooth is stuck and unable to erupt normally, periapical radiographs help assess its position and guide treatment decisions.   **Assessing Dental Trauma:**   - **Fractures:** Both periapical and bitewing radiographs can visualize fractures in the tooth crown or root, aiding in diagnosing the severity of the injury and planning treatment. - **Alveolar Bone Damage:** Periapical radiographs can reveal damage to the jawbone supporting the tooth following trauma.   **Other Diagnostic Uses:**   - **Unexplained Toothache:** When the source of a child's toothache is unclear, periapical radiographs can help identify potential causes like abscesses or hidden caries. - **Root Resorption:** Periapical radiographs can detect root resorption, a condition where the tooth root starts to dissolve, which can be caused by trauma or inflammation.   **Treatment Planning and Monitoring:**   - **Root Canal Treatment:** Periapical radiographs are essential for planning and monitoring root canal treatment in children. - **Orthodontic Treatment:** Depending on the complexity of the orthodontic case, bitewing radiographs may be used to assess tooth alignment and root position.   **When to Weigh the Benefits and Risks:**  While valuable, it's important to remember that X-rays involve radiation exposure. Pediatric dentists carefully consider the necessity of radiographs and prioritize minimizing radiation exposure for children. For minor dental issues, a clinical examination might suffice.  **Conclusion:**  Periapical and bitewing radiographs are crucial tools in pediatric dentistry, offering detailed information for diagnosis, treatment planning, and monitoring various dental conditions in children. However, dentists prioritize using them judiciously, balancing the benefits with the principle of minimizing radiation exposure for young patients. | Detailed imaging with periapical and bitewing radiographs in pediatric dentistry can be beneficial in various clinical situations, including:   1. **Diagnosis of Dental Caries:**    - Bitewing radiographs are essential for detecting interproximal caries (cavities between teeth) that may not be visible during a clinical examination.    - Periapical radiographs help assess the extent of caries lesions, especially those that have progressed close to or into the dental pulp. 2. **Assessment of Dental Trauma:**    - Periapical radiographs can reveal root fractures, displacement of teeth, and other injuries to the teeth and supporting structures following trauma.    - Bitewing radiographs can help detect alveolar bone fractures in the region of the injured teeth. 3. **Evaluation of Dental Development:**    - Periapical radiographs are used to monitor the development of permanent teeth, assess root formation, and detect any abnormalities in tooth eruption patterns.    - Bitewing radiographs can help identify missing or supernumerary teeth (extra teeth). 4. **Diagnosis of Endodontic Pathology:**    - Periapical radiographs are crucial for diagnosing periapical lesions (infections at the root tip), which may not present with obvious clinical symptoms in children. 5. **Monitoring of Orthodontic Treatment:**    - Periapical radiographs can be used to assess root resorption (loss of root structure) during orthodontic treatment.    - Bitewing radiographs can help monitor the progress of tooth movement and identify any potential complications. 6. **Assessment of Periodontal Health:**    - Bitewing radiographs can reveal early signs of periodontal disease, such as bone loss around the teeth, in children and adolescents with risk factors. 7. **Evaluation of Pathological Lesions:**    - Periapical and bitewing radiographs can help identify and assess the extent of cysts, tumors, and other pathological lesions in the jaws.   **Important Considerations:**   - The use of radiographs in pediatric dentistry should be based on individual patient needs and follow the ALARA principle (As Low As Reasonably Achievable). - The frequency and type of radiographs should be determined by the child's age, dental history, clinical findings, and risk factors for dental diseases. - The use of digital radiography and appropriate shielding techniques can further reduce radiation exposure in children.   By employing detailed imaging with periapical and bitewing radiographs judiciously, pediatric dentists can accurately diagnose and effectively manage various dental conditions, ensuring optimal oral health for their young patients | Detailed imaging with periapical and bitewing radiographs can be beneficial in several clinical situations in pediatric dentistry. Here are some scenarios where these radiographs are useful:   1. Caries Detection and Assessment:    - Bitewing Radiographs: Detect interproximal caries (between teeth) in primary and permanent dentition.    - [Periapical Radiographs: Assess caries extent, periapical pathology, and root development^1^](https://link.springer.com/article/10.1007/s40368-019-00493-x). 2. Periodontal Disease Evaluation:    - [Clinical Judgment: Use selected bitewing or periapical images to evaluate periodontal disease beyond nonspecific gingivitis^2^](https://www.aapd.org/assets/1/7/E_Radiographs1.PDF). 3. Dental Infections:    - Periapical Radiographs: Identify periapical pathology, abscesses, or localized infections.    - [Panoramic Radiography (PR): Assess overall dental health, including infections^1^](https://link.springer.com/article/10.1007/s40368-019-00493-x). 4. Dental Trauma:    - Periapical Radiographs: Evaluate traumatic injuries to teeth and supporting structures.    - [PR: Assess trauma-related changes^1^](https://link.springer.com/article/10.1007/s40368-019-00493-x). 5. Dental Anomalies and Developmental Disorders:    - Bitewing and Periapical Radiographs: Reveal anomalies, hypodontia, supernumerary teeth, and developmental disturbances.    - [PR: Provides a broader view of dental arches](https://link.springer.com/article/10.1007/s40368-019-00493-x)[^1^](https://link.springer.com/article/10.1007/s40368-019-00493-x).   [Remember that radiographic decisions should follow the ALADAIP principle (As Low As Diagnostically Achievable, Indication-oriented, and Patient-specific) to minimize radiation exposure while obtaining necessary diagnostic information^1^](https://link.springer.com/article/10.1007/s40368-019-00493-x). | Kühnisch J, Anttonen V, Duggal MS, Spyridonos ML, Rajasekharan S, Sobczak M, Stratigaki E, Van Acker JWG, Aps JKM, Horner K, Tsiklakis K. Best clinical practice guidance for prescribing dental radiographs in children and adolescents: an EAPD policy document. Eur Arch Paediatr Dent. 2020 Aug;21(4):375-386. doi: 10.1007/s40368-019-00493-x. PMID: 31768893 |
|  | **ChatGPT 3.5** | **ChatGPT 4** | **Chat GPT 4o** [**‎**](https://bard.google.com/) | **GEMINI** | **GEMINI ADNANCED** | **COPILOT** | **Evidence (literature)** |
| **4.** What are the recommended features (concentration [ppmF], frequency, and amount) of fluoride toothpaste in the following age groups of children: (1) up to 2 years, (2) 2-6 years, and (3) over 6 years? | The recommended features of fluoride toothpaste, including concentration (ppmF), frequency, and amount, vary based on the age group of children to ensure optimal dental health while minimizing the risk of fluorosis. Here are the recommendations for each age group:   1. **Up to 2 years:**    - **Fluoride Concentration:** The American Academy of Pediatric Dentistry (AAPD) recommends using a smear or rice-sized amount of fluoride toothpaste containing 1,000 ppmF (parts per million of fluoride) for children up to 2 years old.    - **Frequency:** Brushing should occur twice daily, ideally after breakfast and before bedtime.    - **Amount:** A smear or rice-sized amount of toothpaste should be used on the toothbrush. Since young children may not be able to spit out toothpaste effectively, it's important to minimize the amount of toothpaste used to reduce the risk of ingesting excessive fluoride. 2. **2-6 years:**    - **Fluoride Concentration:** For children aged 2 to 6 years, fluoride toothpaste with a concentration of 1,000 ppmF to 1,500 ppmF is recommended.    - **Frequency:** Brushing should occur twice daily, preferably after breakfast and before bedtime.    - **Amount:** A pea-sized amount of fluoride toothpaste should be used on the toothbrush. Children should be encouraged to spit out excess toothpaste after brushing but should avoid rinsing with water immediately afterward to allow the fluoride to remain in contact with the teeth for a longer duration. 3. **Over 6 years:**    - **Fluoride Concentration:** For children over 6 years old, fluoride toothpaste with a concentration of 1,350 ppmF to 1,500 ppmF is recommended.    - **Frequency:** Brushing should occur twice daily, after meals if possible, with special attention to brushing before bedtime.    - **Amount:** A pea-sized amount of fluoride toothpaste should be used on the toothbrush. Children should be encouraged to spit out excess toothpaste after brushing but should avoid rinsing with water immediately afterward to maximize the benefits of fluoride.   It's important for parents and caregivers to supervise children's toothbrushing routines and ensure they use the appropriate amount of fluoride toothpaste based on their age. Additionally, regular dental visits are essential for professional assessment of oral health and personalized recommendations regarding fluoride use and other preventive measures. | Fluoride toothpaste is an effective tool for preventing dental caries in children. The recommendations for fluoride concentration, frequency of use, and amount of toothpaste vary by age group to balance the benefits of caries prevention with the risk of dental fluorosis. Here are the current guidelines:   1. **Children Up to 2 Years**:    - **Concentration**: Use a smear of toothpaste with a lower fluoride concentration (around 1000 ppmF).    - **Frequency**: Brush teeth twice a day.    - **Amount**: A smear or rice-sized amount of toothpaste is recommended to minimize swallowing, which can be significant in this age group. 2. **Children Aged 2-6 Years**:    - **Concentration**: Use a toothpaste with a fluoride concentration of 1000-1350 ppmF.    - **Frequency**: Brush teeth twice a day.    - **Amount**: A pea-sized amount of toothpaste should be used to ensure adequate fluoride exposure while still minimizing the risk of fluorosis from swallowing toothpaste. 3. **Children Over 6 Years**:    - **Concentration**: Toothpaste with a fluoride concentration of 1350-1500 ppmF is appropriate.    - **Frequency**: Brushing should occur twice a day.    - **Amount**: A pea-sized amount remains recommended as children over six are generally better at spitting out toothpaste, but ensuring they do not swallow large amounts is still important.   **Additional Guidelines:**   - **Supervision**: Children under the age of 6 should be supervised during brushing to ensure they use the correct amount of toothpaste and avoid swallowing it. - **Education**: Teach children to spit out toothpaste after brushing and not to rinse with water immediately to maximize the topical benefits of fluoride. - **Dental Visits**: Regular dental visits should be scheduled starting at the eruption of the first tooth or by the child's first birthday to assess risk of caries and discuss appropriate fluoride use.   These guidelines help maximize the benefits of fluoride in preventing dental caries while minimizing the risk of fluorosis, especially important during the years of primary tooth development and when the permanent teeth are erupting. | **Recommended Features of Fluoride Toothpaste for Children by Age Group**  The appropriate use of fluoride toothpaste is crucial for preventing dental caries in children. The recommendations vary by age group to ensure both effectiveness in caries prevention and safety in terms of fluoride ingestion.  **1. Children Up to 2 Years:**   - **Concentration**: Use a fluoride toothpaste with a concentration of **1000 ppm fluoride (ppmF)**. - **Frequency**: Brush **twice a day**. - **Amount**: Use a **smear or rice-sized amount** of toothpaste.   **2. Children Aged 2-6 Years:**   - **Concentration**: Use a fluoride toothpaste with a concentration of **1000-1350 ppm fluoride (ppmF)**. - **Frequency**: Brush **twice a day**. - **Amount**: Use a **pea-sized amount** of toothpaste.   **3. Children Over 6 Years:**   - **Concentration**: Use a fluoride toothpaste with a concentration of **1350-1500 ppm fluoride (ppmF)**. - **Frequency**: Brush **twice a day**. - **Amount**: Use a **pea-sized amount** of toothpaste.   **Additional Recommendations**   - **Supervision**: Parents or caregivers should supervise brushing to ensure the correct amount of toothpaste is used and to minimize swallowing. - **Spitting, Not Rinsing**: Encourage children to spit out the toothpaste after brushing rather than rinsing with water to maximize fluoride contact with the teeth. - **Toothbrush Selection**: Use an age-appropriate toothbrush with soft bristles to avoid damage to the gums and developing teeth.   **Rationale for Recommendations**   - **Up to 2 Years**: Young children are at a higher risk of dental fluorosis due to the potential ingestion of toothpaste. Therefore, a lower concentration of fluoride and a very small amount (smear) are recommended. - **2-6 Years**: As children grow, they can better understand the need to spit out toothpaste, allowing for a slightly higher concentration and amount, while still being cautious of the risk of fluorosis. - **Over 6 Years**: Older children can use the same concentration of fluoride toothpaste as adults. They are more capable of spitting out toothpaste, thus reducing the risk of fluoride ingestion.   **Summary**  Using the recommended concentration, frequency, and amount of fluoride toothpaste according to age ensures effective caries prevention while minimizing the risk of dental fluorosis. Supervising young children during brushing and encouraging proper brushing techniques are key components of maintaining optimal oral health. | The American Dental Association (ADA) recommends different fluoride toothpaste features for various age groups to ensure safety and effectiveness:   \| **Age Group** \| **Fluoride Concentration (ppmF)** \| **Brushing Frequency** \| **Amount** \| \| --- \| --- \| --- \| --- \| \| Up to 2 years \| 0 ppmF \| Not recommended \| Not applicable \| \| 2-6 years \| 500 ppmF \| Supervised use, pea-sized amount \| Pea-sized amount \| \| Over 6 years \| 1000 ppmF \| Supervised or unsupervised use, pea-sized amount \| Pea-sized amount \|  drive_spreadsheetHere's a breakdown of the reasoning behind these recommendations:**Up to 2 years:** Children this young are still developing swallowing habits and may ingest a significant amount of toothpaste. Since they're not spitting effectively yet, using fluoride-free toothpaste is recommended to minimize potential fluoride overexposure.**2-6 years:** This age group benefits from the cavity-fighting properties of fluoride toothpaste, but supervision is crucial to ensure they don't swallow a large amount. A pea-sized amount of low-fluoride (500 ppmF) toothpaste is recommended.**Over 6 years:** By this age, most children can spit more effectively, reducing the risk of swallowing toothpaste. Higher fluoride concentration (1000 ppmF) becomes appropriate to provide optimal protection against cavities. However, supervision is still recommended for some children in this age group.**Additional Tips:**Look for toothpaste with the ADA Seal of Acceptance to ensure it meets safety and effectiveness standards.Regardless of age, always supervise young children while brushing to ensure they use a pea-sized amount and spit properly.If you have concerns about fluorosis (excessive fluoride intake causing white spots on teeth), consult your child's dentist. They can advise on the appropriate fluoride concentration for your child's individual needs. | Fluoride Toothpaste Recommendations for Children:  \| **Age Group** \| **Fluoride Concentration (ppmF)** \| **Brushing Frequency** \| **Amount of Toothpaste** \| \| --- \| --- \| --- \| --- \| \| Up to 2 years \| 1000 \| Smear (rice grain size), after the first tooth erupts \| Supervised brushing \| \| 2-6 years \| 1000 (up to 1500) \| Pea-sized amount, twice daily \| Supervised brushing \| \| Over 6 years \| 1450 (up to 1500) \| Pea-sized amount, twice daily \| Twice daily \|  **Key Points:****Up to 2 years:** Fluoridated toothpaste is recommended as soon as the first tooth erupts. Use a smear (rice grain size) and supervise brushing to minimize the amount swallowed.**2-6 years:** A pea-sized amount of fluoridated toothpaste is recommended. Supervise brushing to ensure children spit out the toothpaste and don't swallow it.**Over 6 years:** Children can use a pea-sized amount of fluoridated toothpaste twice daily. Encourage them to spit out the toothpaste after brushing.**Important Considerations:**The exact fluoride concentration (ppmF) might vary slightly depending on the brand and specific product.These are general recommendations, and your dentist may provide specific advice based on your child's individual needs and risk factors.By following these recommendations, you can help ensure your child receives the right amount of fluoride to protect their teeth from decay while minimizing the risk of fluorosis (excess fluoride intake). | Here are the **recommended features** for fluoride toothpaste use in different age groups of children:**Up to 2 years**:**Fluoride Concentration**: Begin using fluoride toothpaste at age 2 years.**Amount**: Use a smear the size of a **rice grain**.[**Frequency**: Brush teeth at least twice daily^1^](https://www.cdc.gov/mmwr/volumes/68/wr/mm6804a3.htm).**2-6 years**:**Fluoride Concentration**: Continue using no more than a **pea-sized amount** (0.25 g).**Frequency**: Brush teeth at least twice daily.[**Prevent Ingestion**: Ensure parental supervision to prevent inadvertent swallowing^1^](https://www.cdc.gov/mmwr/volumes/68/wr/mm6804a3.htm).**Over 6 years**:**Fluoride Concentration**: Continue with a **pea-sized amount**.**Frequency**: Brush teeth at least twice daily.[**Swallowing Reflex**: By age 6, the swallowing reflex is developed sufficiently to prevent accidental ingestion^1^](https://www.cdc.gov/mmwr/volumes/68/wr/mm6804a3.htm).Remember, proper toothpaste use contributes to oral health and cavity prevention! | Toumba KJ, Twetman S, Splieth C, Parnell C, van Loveren C, Lygidakis NΑ. Guidelines on the use of fluoride for caries prevention in children: an updated EAPD policy document. Eur Arch Paediatr Dent. 2019 Dec;20(6):507-516. doi: 10.1007/s40368-019-00464-2. PMID: 31631242 |
|  | **ChatGPT 3.5** | **ChatGPT 4** | **Chat GPT 4o** [**‎**](https://bard.google.com/) | **GEMINI** | **GEMINI ADNANCED** | **COPILOT** | **Evidence (literature)** |
| **5.** What is the clinical effectiveness of different restorative materials (amalgam, conventional glass‑ionomer cements, metal-reinforced glass‑ionomer cements, resin-modified glass-ionomer cements, high viscosity glass-ionomer cements, compomer, composite resin) for the class II cavity restorations in a carious primary teeth. | The clinical effectiveness of different restorative materials for class II cavity restorations in carious primary teeth can vary based on factors such as longevity, retention, aesthetics, ease of placement, and biocompatibility. Here's a general overview of the clinical effectiveness of some commonly used restorative materials:   1. **Amalgam**: Amalgam has been traditionally used for class II restorations due to its durability, longevity, and cost-effectiveness. However, its use has declined in recent years due to concerns about mercury content and aesthetics. 2. **Conventional Glass-Ionomer Cements (GICs):** Conventional GICs have good adhesion to tooth structure and release fluoride, which can help prevent secondary caries. However, they are relatively weak compared to other materials and may have limited longevity in high-stress areas. 3. **Metal-Reinforced Glass-Ionomer Cements:** These materials combine the benefits of conventional GICs with increased strength due to metal reinforcement. They offer better durability and wear resistance compared to conventional GICs. 4. **Resin-Modified Glass-Ionomer Cements (RM-GICs):** RM-GICs have improved physical properties compared to conventional GICs, including better strength and wear resistance. They also bond chemically to tooth structure and can be more aesthetically pleasing. 5. **High Viscosity Glass-Ionomer Cements:** High viscosity GICs are similar to conventional GICs but have a thicker consistency, which may make them easier to handle and place in deep cavities. They provide good fluoride release and adhesion to tooth structure. 6. **Compomer**: Compomers are a hybrid material combining the benefits of composites and glass-ionomers. They offer improved aesthetics compared to conventional GICs but may not be as durable or long-lasting as composite resins. 7. **Composite Resin:** Composite resins are widely used for their excellent aesthetics and ability to bond to tooth structure, providing a strong and durable restoration. They offer superior aesthetics and wear resistance compared to most other materials but may require more technique-sensitive placement.   The choice of restorative material depends on various factors, including the patient's age, oral health status, cavity size and location, aesthetic demands, and clinician preference. A comprehensive evaluation of these factors should be done to select the most appropriate material for each case. Long-term clinical studies comparing the performance of these materials specifically in primary teeth are essential for providing evidence-based recommendations. | To assess the clinical effectiveness of different restorative materials for Class II cavity restorations in carious primary teeth, we can look at various aspects such as longevity, resistance to secondary caries, and maintenance of tooth integrity. Here's a brief overview of each material:   1. **Amalgam**: Known for its strength and longevity, amalgam is less popular in pediatric dentistry due to aesthetic concerns and mercury content. However, it performs well in terms of durability and wear resistance. 2. **Conventional Glass-Ionomer Cements (GIC)**: These materials release fluoride which can help in preventing secondary caries. They bond chemically to dental tissue but have lower fracture toughness compared to amalgam and composite resins. 3. **Metal-Reinforced Glass-Ionomer Cements**: These are stronger than conventional GICs due to the addition of metal particles. They offer better mechanical properties and are more resistant to wear. 4. **Resin-Modified Glass-Ionomer Cements (RMGIC)**: These materials combine the fluoride release and chemical bond of GICs with improved aesthetics and strength from the resin component. They are more resistant to moisture degradation compared to conventional GICs. 5. **High Viscosity Glass-Ionomer Cements**: Specifically designed for load-bearing areas, these cements are more resistant to wear and have higher fracture toughness. They are suitable for areas subjected to masticatory forces. 6. **Compomer**: These materials are hybrids between composite resins and glass ionomers. They offer better aesthetics than GICs and are easier to handle but may not provide the same level of fluoride release. 7. **Composite Resin**: Offers the best aesthetics and is highly preferred for visible areas. Composite resins have good wear resistance and strength but do not release fluoride, which could prevent further caries.   Each material has its advantages and disadvantages depending on the clinical situation, patient's age, tooth position, and caries risk. For example, for high caries risk patients, a fluoride-releasing material like glass ionomer or RMGIC might be preferred. In terms of aesthetics, composite resins or compomers would be more suitable.  Long-term studies and systematic reviews would provide the best evidence for comparing these materials under various clinical conditions. | To address this question, I will look up recent studies and reviews on the clinical effectiveness of these restorative materials for class II cavity restorations in primary teeth. This will include comparing their performance, longevity, failure rates, and any other relevant clinical outcomes. Please hold on while I gather the information. ​​  The clinical effectiveness of different restorative materials for class II cavity restorations in carious primary teeth has been widely studied. Here is a summary of the findings for various materials:   1. **Amalgam**: Amalgam restorations have been traditionally used and are known for their durability and low failure rates. Studies have shown that amalgam has one of the lowest mean failure rates at 24 months compared to other materials​ ([MDPI](https://www.mdpi.com/2077-0383/11/12/3490))​​ ([JOCPD](https://www.jocpd.com/articles/10.17796/1053-4628-39.4.315))​. 2. **Conventional Glass-Ionomer Cements (GIC)**: Conventional GICs are less durable compared to resin-based composites and amalgam, often showing higher failure rates. However, they are beneficial due to their fluoride release, which can help prevent secondary caries​ ([MDPI](https://www.mdpi.com/2077-0383/11/12/3490))​. 3. **Metal-Reinforced Glass-Ionomer Cements**: These materials have been found to have higher failure rates compared to amalgam and composite resins. Their usage is less common due to these higher failure rates and less favorable clinical outcomes​ ([MDPI](https://www.mdpi.com/2077-0383/11/12/3490))​. 4. **Resin-Modified Glass-Ionomer Cements (RMGIC)**: RMGICs offer better durability than conventional GICs and also release fluoride. They are considered a good option for primary teeth restorations, balancing durability with additional caries protection​ ([JOCPD](https://www.jocpd.com/articles/10.17796/1053-4628-39.4.315))​. 5. **High Viscosity Glass-Ionomer Cements**: These materials tend to have higher failure rates over time, particularly noted at 36 months. They are not as durable as composite resins or amalgam but are used for their ease of application and fluoride release​ ([MDPI](https://www.mdpi.com/2077-0383/11/12/3490))​. 6. **Compomer**: Compomers show a performance similar to resin composites, with relatively low failure rates. They combine the benefits of composites and glass ionomers, making them a favorable choice for pediatric restorations​ ([JOCPD](https://www.jocpd.com/articles/10.17796/1053-4628-39.4.315))​. 7. **Composite Resin**: Composite resins are highly popular due to their aesthetic qualities and good durability. They have one of the lowest failure rates at both 24 and 36 months, making them an effective material for class II restorations in primary teeth​ ([MDPI](https://www.mdpi.com/2077-0383/11/12/3490))​.   In summary, while amalgam and composite resins generally show the best performance in terms of longevity and durability, other materials like RMGICs and compomers provide additional benefits like fluoride release, which can be crucial in pediatric dentistry for preventing further caries. The choice of material often depends on specific clinical scenarios, patient needs, and practitioner preference. | Several restorative materials are used for class II cavities in primary teeth, each with varying degrees of effectiveness:   - **Amalgam:** Traditionally used, amalgam shows annual failure rates between 1-28% [3]. However, its use is declining due to aesthetic concerns and environmental factors. - **Glass-ionomer cements (GICs):** These offer fluoride release and good biocompatibility but may have lower wear resistance. Annual failure rates vary depending on the type:   - Conventional GICs: 7.6-16.6% [3].   - Metal-reinforced GICs: Not ideal due to high failure rates around 29.9% [3].   - Resin-modified GICs (RMGICs): A good balance, with failure rates of 1.9-16.9% [3].   - High-viscosity GICs: Similar to RMGICs, with failure rates between 2.9-25.6% [3]. - **Compomers:** These combine the properties of GICs and composites. They show promise, with failure rates between 0-14.7% [3]. - **Composite resins:** Popular for aesthetics, their success relies on proper bonding and technique. Annual failure rates range from 0-19.5% [3]. Bulk-fill composites might be a promising option as well, with failure rates of 0-16.9% [3].   **Overall, Resin-modified glass-ionomer cements (RMGICs) seem to offer a good balance of clinical effectiveness for class II restorations in primary teeth.** They provide fluoride release, acceptable wear resistance, and good bonding properties.  Here are some additional points to consider:   - This information is based on annual failure rates. Longevity can vary depending on factors like child's age, oral hygiene, and dentist's skill. - Consult a dentist for the best choice of restorative material based on the specific situation of the child's tooth.   For further details, you can search for systematic reviews on clinical effectiveness of restorative materials in primary teeth. | The clinical effectiveness of various restorative materials for Class II cavity restorations in primary teeth has been studied extensively, with varying results depending on the material and the specific outcome measured. Here's a summary of the available evidence:   - **Amalgam:** Historically considered the gold standard due to its durability and longevity, amalgam has shown good clinical success rates in primary teeth. However, its use is declining due to concerns about mercury content and aesthetics. - **Conventional Glass-ionomer Cements (GIC):** GICs offer advantages like fluoride release and chemical adhesion to tooth structure but are less durable than amalgam or resin composites, particularly in load-bearing restorations like Class II cavities. - **Metal-reinforced GIC:** Adding metal particles to GICs improves their strength and wear resistance. However, they may not be as durable as amalgam or resin composites in the long term. - **Resin-modified GIC (RMGIC):** RMGICs combine the fluoride release of GICs with improved aesthetics and handling properties compared to conventional GICs. They generally perform better than GICs but may still not be as durable as resin composites. - **High-viscosity GIC (HV-GIC):** HV-GICs offer higher viscosity and better wear resistance than conventional GICs, making them potentially more suitable for Class II restorations. However, their clinical performance data are limited. - **Compomer:** Compomers are a hybrid material containing both glass-ionomer and resin components. They generally perform better than conventional GICs but may not match the durability of resin composites. - **Composite Resin:** Composite resins are popular for their aesthetics and ability to bond to tooth structure. They can provide excellent clinical outcomes in Class II restorations if placed properly, but their longevity may be affected by factors like polymerization shrinkage and moisture contamination.   **Overall:**  While there's no single "best" material for all cases, several studies suggest that resin composites and amalgam generally offer the highest survival rates in Class II restorations in primary teeth. However, the choice of material should consider individual patient factors like caries risk, age, and parental preferences.  **Important Considerations:**   - **Caries risk:** High-caries-risk children may benefit from materials with fluoride release, like GICs, RMGICs, or compomers. - **Child's age:** Restorations in younger children may be more prone to failure due to factors like behavioral challenges and shorter remaining tooth life. - **Parental preferences:** Some parents may prefer avoiding amalgam due to concerns about mercury.   It's crucial to consult with a pediatric dentist to determine the most appropriate restorative material for each individual case based on a thorough assessment of the child's oral health and specific needs.  For further information, you can refer to the following research articles:   - **Clinical effectiveness of restorative materials for the restoration of carious primary teeth without pulp therapy: a systematic review**   Please note that this information is not a substitute for professional dental advice. | [An **umbrella review** published in the *Journal of Clinical Medicine*^1^](https://www.mdpi.com/2077-0383/11/12/3490) assessed the quality of evidence from systematic reviews on this topic. Here are the key findings:   1. **Amalgam**: Amalgam performed well and had the **lowest mean failure rate** at **24 months**. 2. **Resin Composite**: Resin composite also had a low failure rate at 24 months. 3. **High-Viscosity Glass Ionomer Cements**: These showed the **highest failure rate** at 36 months. 4. **Metal-Reinforced Glass Ionomer Cements**: Similar to high-viscosity glass ionomer cements, they had a higher failure rate. 5. **Compomer**: Compomer had the **lowest failure rate** at 36 months.   Overall, all materials studied were **equally efficient** for restoring carious primary teeth. Keep in mind that individual patient factors and clinical context may influence material selection. [For more detailed information, you can refer to the full article here](https://doi.org/10.3390/jcm11123490)[^1^](https://www.mdpi.com/2077-0383/11/12/3490). | Amend S, Boutsiouki C, Bekes K, Kloukos D, Lygidakis NN, Frankenberger R, Krämer N. Clinical effectiveness of restorative materials for the restoration of carious primary teeth without pulp therapy: a systematic review. Eur Arch Paediatr Dent. 2022 Oct;23(5):727-759. doi: 10.1007/s40368-022-00725-7. PMID: 35819627 |
|  | **ChatGPT 3.5** | **ChatGPT 4** | **Chat GPT 4o** [**‎**](https://bard.google.com/) | **GEMINI** | **GEMINI ADNANCED** | **COPILOT** | **Evidence (literature)** |
| **6.** Treating deep carious lesions in primary teeth using pulpotomy, which medicament is best: Mineral Trioxide Aggregate (MTA), ferric sulfate (FS), formocresol (FC), or calcium hydroxide? What type of restoration should be used after pulpotomy? | When performing a pulpotomy procedure for deep carious lesions in primary teeth, the choice of medicament and restoration depends on various factors including the extent of pulpal involvement, clinical judgment, and the desired outcome. Here's a comparison of the commonly used medicaments and restoration materials for pulpotomy in primary teeth:   1. **Medicaments:**   a. **Mineral Trioxide Aggregate (MTA):**   - - MTA is considered one of the most preferred medicaments for pulpotomy in primary teeth due to its excellent sealing ability, biocompatibility, and ability to stimulate dentin formation.   - It has shown comparable or superior outcomes compared to other medicaments in terms of clinical success rates and long-term outcomes.   b. **Ferric Sulfate (FS):**   - - Ferric sulfate is a hemostatic agent with mild pulpal tissue-fixing properties. It helps control bleeding during pulpotomy procedures.   - While FS has been used historically for pulpotomy, its use has declined in favor of other materials like MTA due to concerns about potential tissue toxicity and inferior outcomes.   c. **Formocresol (FC):**   - - Formocresol is an effective pulpotomy medicament with bactericidal properties. However, its use has decreased due to concerns about potential carcinogenicity, mutagenicity, and tissue toxicity.   - Most dental organizations and experts no longer recommend the routine use of formocresol for pulpotomy in primary teeth, especially considering the availability of safer and equally effective alternatives like MTA.   d. **Calcium Hydroxide:**   - - Calcium hydroxide has been used historically for pulpotomy in primary teeth. It has antimicrobial properties and promotes the formation of a hard tissue barrier over time.   - While calcium hydroxide has been effective in some cases, its use has decreased in favor of materials like MTA, which offer superior sealing ability and biocompatibility. | Treating deep carious lesions in primary teeth via pulpotomy involves the removal of the inflamed pulp tissue followed by the placement of a medicament to preserve the vitality of the remaining pulp tissue. The choice of medicament depends on various factors including efficacy, biocompatibility, and long-term outcomes. Here’s a detailed overview of the medicaments and the appropriate type of restoration to use after pulpotomy:  **Pulpotomy Medicaments**   1. **Mineral Trioxide Aggregate (MTA)**:    - **Advantages**: MTA is highly biocompatible, promotes healing and hard tissue barrier formation, and has excellent sealing properties, which reduce the risk of microleakage. It is less likely to cause inflammation.    - **Clinical Outcomes**: MTA is considered the gold standard for pulpotomy treatments due to its superior clinical and radiographic success rates. 2. **Ferric Sulfate (FS)**:    - **Advantages**: FS is an astringent that controls bleeding by agglutinating blood proteins to form a mechanical barrier. It is easy to use and has shown good clinical outcomes.    - **Drawbacks**: FS does not promote healing or dentin regeneration like MTA. It acts primarily as a hemostatic agent. 3. **Formocresol (FC)**:    - **Advantages**: Historically, FC was widely used due to its bactericidal and fixative properties.    - **Drawbacks**: Due to potential toxicity, mutagenicity, and allergenicity, the use of FC has declined significantly and is no longer recommended in many countries. 4. **Calcium Hydroxide**:    - **Advantages**: Known for its ability to stimulate dentin formation and antibacterial properties.    - **Drawbacks**: Inferior in forming a consistent dentin bridge and more prone to resorption; thus, it is less favorable compared to MTA for pulpotomy in primary teeth.   **Recommended Medicament**  **MTA** is recommended as the best medicament for pulpotomy in primary teeth due to its high success rates, biocompatibility, and ability to promote tissue regeneration. | **Pulpotomy in Primary Teeth: Best Medicament and Restoration**  **Pulpotomy** is a common procedure for treating deep carious lesions in primary teeth when the pulp is exposed but the radicular pulp is healthy and vital. The choice of medicament and restoration plays a crucial role in the success of the treatment.  **Best Medicament for Pulpotomy**  Several medicaments have been traditionally used in pulpotomy, each with its advantages and limitations. Here is a comparison of the commonly used medicaments:   1. **Mineral Trioxide Aggregate (MTA)**:    - **Advantages**: MTA is highly biocompatible, promotes hard tissue formation, and has excellent sealing properties. It has a high success rate and encourages the formation of a dentin bridge.    - **Disadvantages**: It is more expensive and has a longer setting time compared to other materials.    - **Best Use**: MTA is considered the gold standard for pulpotomy in primary teeth due to its superior outcomes and biocompatibility. 2. **Ferric Sulfate (FS)**:    - **Advantages**: Ferric sulfate is hemostatic, easy to use, and has a comparable success rate to MTA.    - **Disadvantages**: It does not promote dentin bridge formation and may cause internal resorption if not used properly.    - **Best Use**: FS is a good alternative to MTA, especially when cost is a concern. 3. **Formocresol (FC)**:    - **Advantages**: Formocresol has been traditionally used with a high success rate.    - **Disadvantages**: Concerns over its potential cytotoxicity, mutagenicity, and carcinogenicity have led to reduced use in favor of safer alternatives.    - **Best Use**: FC is less favored due to safety concerns, despite its effectiveness. 4. **Calcium Hydroxide**:    - **Advantages**: Calcium hydroxide has antibacterial properties and promotes the formation of a hard tissue barrier.    - **Disadvantages**: It is less effective in maintaining long-term pulp vitality and is associated with internal resorption and poor success rates compared to MTA and FS.    - **Best Use**: Calcium hydroxide is generally not preferred for pulpotomy in primary teeth due to its lower success rates.   **Conclusion**: **Mineral Trioxide Aggregate (MTA)** is the best medicament for pulpotomy in primary teeth due to its superior biocompatibility, sealing ability, and long-term success rates. **Ferric Sulfate (FS)** is a good alternative when cost is an issue, while **Formocresol (FC)** is less recommended due to safety concerns. **Calcium Hydroxide** is generally not recommended due to its lower success rate. | Among the medicaments you mentioned for pulpotomy in primary teeth, there's a shift towards newer options due to some drawbacks of the traditional materials:   - **Mineral Trioxide Aggregate (MTA):** Currently considered the **gold standard** for pulpotomy in primary teeth. Here's why:   - **Biocompatible:** Minimizes risk of inflammation or allergic reactions.   - **Promotes Healing:** Stimulates dentin bridge formation, sealing the exposed pulp and promoting healing.   - **Bacterial Control:** Has some antibacterial properties.   - **Faster Treatment:** May allow for placement of a permanent restoration in a single visit compared to calcium hydroxide, which often requires a second visit. - **Ferric Sulfate (FS):** A viable alternative with good success rates, particularly for molars. However, it might not be as readily available as MTA and may have a slightly higher chance of internal resorption (gradual breakdown of the inner tooth structure). - **Formocresol (FC):** Previously a widely used pulpotomy medicament, its use is declining due to concerns about:   - **Tissue Fixation:** While effective in killing bacteria, it also fixes surrounding tissues which can hinder healing and complicate future procedures.   - **Potential Toxicity:** Formaldehyde, a component of FC, raises some concerns about potential toxicity, although research on its safety in pulpotomy is inconclusive. - **Calcium Hydroxide (Ca(OH)2):** A traditional pulpotomy medicament, but with limitations:   - **Slower Healing:** Requires a second visit to place the permanent restoration after allowing time for dentin bridge formation.   - **Risk of Discoloration:** Can cause slight discoloration of the tooth. | In pulpotomy of primary teeth, the ideal medicament should be biocompatible, promote healing, and have antimicrobial properties. While all the options you listed have been used, the current consensus favors Mineral Trioxide Aggregate (MTA).   - **Mineral Trioxide Aggregate (MTA):** This material has gained popularity due to its excellent biocompatibility, ability to seal the pulp chamber, and promote dentin bridge formation. Studies have shown high success rates with MTA, making it the preferred choice for many clinicians. - **Ferric Sulfate (FS):** Ferric sulfate is a hemostatic agent that arrests bleeding quickly. While it has been used successfully in pulpotomies, some studies suggest it may not be as effective as MTA in promoting pulp healing and dentin bridge formation. - **Formocresol (FC):** Formocresol was widely used in the past due to its effectiveness in mummifying the pulp tissue. However, concerns about its toxicity and potential systemic effects have led to a decline in its use, especially in primary teeth. - **Calcium Hydroxide:** Calcium hydroxide has been a traditional choice for pulpotomies due to its biocompatibility and ability to stimulate dentin bridge formation. However, its success rates are generally lower than MTA, and it may not be as effective in controlling inflammation. | **Pulpotomy** is a common treatment for deep carious lesions in primary teeth. Let’s explore the available evidence on medicaments and restoration options:   1. **Medicaments for Pulpotomy**:    - [**Mineral Trioxide Aggregate (MTA)** and **formocresol** are recommended based on moderate-quality evidence at 24 months^1^](https://link.springer.com/article/10.1007/s40368-022-00718-6).    - [**Ferric sulfate**, **lasers**, **sodium hypochlorite**, and **tricalcium silicate** are also used, but with weaker evidence^2^](https://www.aapd.org/media/policies_guidelines/g_vpt.pdf).    - [**Calcium hydroxide** is **not** recommended as a pulpotomy medicament for primary teeth with deep caries lesions^2^](https://www.aapd.org/media/policies_guidelines/g_vpt.pdf). | Duggal M, Gizani S, Albadri S, Krämer N, Stratigaki E, Tong HJ, Seremidi K, Kloukos D, BaniHani A, Santamaría RM, Hu S, Maden M, Amend S, Boutsiouki C, Bekes K, Lygidakis N, Frankenberger R, Monteiro J, Anttonnen V, Leith R, Sobczak M, Rajasekharan S, Parekh S. Best clinical practice guidance for treating deep carious lesions in primary teeth: an EAPD policy document. Eur Arch Paediatr Dent. 2022 Oct;23(5):659-666. doi: 10.1007/s40368-022-00718-6. PMID: 36219336.  Stratigaki E, Tong HJ, Seremidi K, Kloukos D, Duggal M, Gizani S. Contemporary management of deep caries in primary teeth: a systematic review and meta-analysis. Eur Arch Paediatr Dent. 2022 Oct;23(5):695-725. doi: 10.1007/s40368-021-00666-7. PMID: 34981447 |
|  | **ChatGPT 3.5** | **ChatGPT 4** | **Chat GPT 4o** [**‎**](https://bard.google.com/) | **GEMINI** | **GEMINI ADNANCED** | **COPILOT** | **Evidence (literature)** |
| **7.** What are the indications, contraindications and adverse effects of using Nitrous oxide/Oxygen sedation in paediatric dental patients | Nitrous oxide/oxygen (N2O/O2) sedation, commonly known as laughing gas, is frequently used in pediatric dentistry for its effectiveness in reducing anxiety and pain during dental procedures. Here are the indications, contraindications, and potential adverse effects associated with its use in pediatric dental patients:  **Indications:**   1. **Anxiety management**: Nitrous oxide is effective in reducing anxiety and apprehension in pediatric patients, making dental treatment more tolerable. 2. **Pain management**: It can help alleviate pain and discomfort during dental procedures. 3. **Behavior management**: Nitrous oxide can aid in behavior management by calming and relaxing pediatric patients, making them more cooperative during treatment. 4. **Control of gag reflex**: It can help suppress the gag reflex, facilitating easier dental procedures.   **Contraindications:**   1. **Nasal obstruction**: Patients with significant nasal obstruction may not receive adequate ventilation with nitrous oxide and are therefore contraindicated. 2. **Respiratory conditions**: Patients with respiratory conditions such as chronic obstructive pulmonary disease (COPD), severe asthma, or bronchitis may be at risk of respiratory depression and should not receive nitrous oxide sedation. 3. **Middle ear infection**: Nitrous oxide can increase middle ear pressure, causing discomfort or complications in patients with an active middle ear infection. 4. **Vitamin B12 deficiency**: Prolonged use of nitrous oxide can inhibit the activity of vitamin B12, leading to potential neurological complications, making it contraindicated in patients with known or suspected vitamin B12 deficiency. 5. **Pregnancy**: Nitrous oxide is contraindicated in pregnant patients, including pregnant dental staff or caregivers.   **Adverse Effects:**   1. **Nausea and vomiting**: Some patients may experience nausea or vomiting during or after nitrous oxide sedation. 2. **Sedation-related complications**: Although rare, complications such as respiratory depression, hypoxia, or aspiration can occur, particularly in patients with pre-existing medical conditions or if the sedation is administered improperly. 3. **Allergic reactions**: Although extremely rare, allergic reactions to nitrous oxide or its components can occur, manifesting as skin rashes, itching, or respiratory symptoms. 4. **Dizziness or disorientation**: Patients may experience dizziness or disorientation during or after nitrous oxide sedation. 5. **Headache**: Headaches are a potential side effect of nitrous oxide use, although they are usually mild and transient.   It's essential for pediatric dentists to carefully assess each patient's medical history and individual needs before administering nitrous oxide sedation, ensuring that the benefits outweigh the risks. Proper monitoring and supervision during sedation are crucial to ensure patient safety and minimize adverse effects. | Nitrous oxide/oxygen sedation, often referred to as inhalation sedation or "laughing gas," is commonly used in pediatric dentistry to manage anxiety and pain. Here's an overview of its indications, contraindications, and potential adverse effects:  **Indications**   1. **Anxiety and Fear**: Used to alleviate anxiety and fear in children undergoing dental procedures. 2. **Mild to Moderate Pain**: Helps in pain management during mildly painful procedures. 3. **Gag Reflex**: Useful in patients with a severe gag reflex that interferes with dental treatment. 4. **Special Healthcare Needs**: Beneficial for patients with special healthcare needs that might not tolerate dental treatment otherwise. 5. **Long Procedures**: Helps in keeping children calm and cooperative during longer dental procedures.   **Contraindications**   1. **Nasal Congestion**: Ineffective in patients who cannot breathe through their nose due to congestion, as nitrous oxide is administered nasally. 2. **Respiratory Conditions**: Should be used with caution or avoided in children with respiratory issues such as chronic obstructive pulmonary disease (COPD) or severe asthma, as it may exacerbate the condition. 3. **Emotional Instability or Behavioral Disorders**: May not be suitable for children who cannot understand or cooperate with the use of a nasal mask. 4. **Certain Medical Conditions**: Conditions like multiple sclerosis, cobalamin deficiency, and some forms of myasthenia gravis may be exacerbated by nitrous oxide. 5. **First Trimester of Pregnancy**: Typically avoided during the first trimester of pregnancy due to potential risks, although this is more relevant for staff and potentially for teenage patients.   **Adverse Effects**   1. **Nausea and Vomiting**: The most common adverse effect, which can be minimized by reducing the concentration of nitrous oxide towards the end of the session. 2. **Diffusion Hypoxia**: A rare phenomenon that can occur if oxygen is not administered for at least 5 minutes after discontinuing nitrous oxide, leading to headache, lethargy, or feeling faint. 3. **Euphoria**: While often considered a beneficial effect, excessive euphoria can sometimes lead to behavioral control issues during the procedure. 4. **Dizziness and Light-headedness**: Some children might experience these symptoms during or after sedation. 5. **Allergic Reactions**: Extremely rare since nitrous oxide is generally considered non-allergenic.   Proper assessment and precaution are crucial when opting for nitrous oxide/oxygen sedation in pediatric patients. It requires careful consideration of the patient's medical history, current health status, and the nature of the dental procedure. Using the lowest effective concentration that provides therapeutic effects while monitoring the child throughout the procedure can help mitigate risks. | To address this question, I'll summarize the indications, contraindications, and adverse effects of using nitrous oxide/oxygen sedation in pediatric dental patients based on current guidelines and literature.  **Indications:**   1. **Anxiety and Fear**: Useful for children who experience significant anxiety or fear during dental procedures. 2. **Gag Reflex**: Helps manage patients with a hyperactive gag reflex. 3. **Lengthy Procedures**: Suitable for lengthy or more complex dental treatments to improve patient cooperation. 4. **Special Needs**: Beneficial for patients with special needs who may have difficulty cooperating with dental treatment. 5. **Need for Relaxation**: Helps to relax the patient without putting them to sleep, allowing them to remain conscious and responsive.   **Contraindications:**   1. **Respiratory Issues**: Not recommended for patients with severe respiratory conditions such as chronic obstructive pulmonary disease (COPD) or severe asthma. 2. **Recent ENT Infections**: Upper respiratory tract infections, middle ear disturbances, or sinusitis may be aggravated. 3. **Psychiatric Disorders**: Patients with certain psychiatric conditions, particularly those with a history of psychosis, may have an adverse reaction. 4. **Vitamin B12 Deficiency**: Chronic exposure can lead to vitamin B12 deficiency. 5. **Inability to Breathe Through the Nose**: Effective administration requires nasal breathing, making it unsuitable for patients with nasal obstructions.   **Adverse Effects:**   1. **Nausea and Vomiting**: Most common adverse effect, especially with higher concentrations or prolonged exposure. 2. **Headache**: May occur post-operatively, often related to inadequate oxygenation during recovery. 3. **Dizziness**: A transient effect during or after the procedure. 4. **Shivering**: May occur during the recovery phase. 5. **Behavioral Effects**: Rarely, children may experience excitement or uncooperative behavior.   Nitrous oxide/oxygen sedation is generally considered safe and effective for managing anxiety and discomfort in pediatric dental patients when used appropriately. It is crucial to have a thorough patient assessment and monitoring during and after the procedure to mitigate potential risks. | Nitrous oxide/oxygen sedation, also known as laughing gas, is a common technique used to manage anxiety and discomfort during dental procedures in children.  Here's a breakdown of its use:  **Indications:**   - **Anxiety and Fear:** Nitrous oxide is excellent for calming anxious or fearful children, making treatment easier and more pleasant. - **Special Needs Patients:** Children with special needs who may struggle to cooperate can benefit from nitrous oxide to facilitate dental care. - **Strong Gag Reflex:** A strong gag reflex can hinder dental procedures. Nitrous oxide can help relax the patient and minimize gagging. - **Limited Local Anesthesia:** In some cases, local anesthesia may not be fully effective. Nitrous oxide can provide additional pain relief and relaxation. - **Unpleasant or Lengthy Procedures:** For longer or more complex procedures, nitrous oxide can improve the child's tolerance and comfort level.   **Contraindications:**   - **Respiratory Issues:** Children with upper respiratory infections, allergies causing nasal congestion, or recent middle ear surgery are not good candidates due to potential breathing difficulties. - **Gastrointestinal Issues:** Nitrous oxide can increase nausea and vomiting, so it's avoided in children with gastrointestinal problems like recent bowel obstruction (ileus). - **Neurological Conditions:** Certain neurological conditions or recent head injuries may be contraindications. - **Mental Health Concerns:** Children with severe mental health issues or inability to follow instructions might not be suitable for nitrous oxide sedation. - **Substance Abuse:** A history of substance abuse could be a contraindication.   **Adverse Effects:**  Nitrous oxide is generally safe when administered properly by trained professionals. However, some mild adverse effects can occur:   - **Nausea and Vomiting:** The most common side effect, usually minimal and resolves quickly after stopping the nitrous oxide. - **Lightheadedness and Dizziness:** May occur briefly during or after administration. - **Tingling:** Some children may experience tingling in their hands or feet. - **Ear Pressure:** Nitrous oxide can cause a feeling of ear pressure, similar to airplane travel.   **Overall, nitrous oxide/oxygen sedation is a safe and effective technique for managing anxiety and discomfort in pediatric dentistry. A dentist will discuss any potential risks and benefits based on the individual child's health and needs.** | Nitrous oxide/Oxygen sedation (N2O/O2) is a safe and effective method for managing anxiety and pain in pediatric dental patients. However, it's important to understand its indications, contraindications, and potential adverse effects.  **Indications:**   - **Anxious or fearful patients:** N2O/O2 helps to reduce anxiety and fear, making dental procedures more tolerable for children. - **Patients with special needs:** N2O/O2 can be helpful for children with certain medical or developmental conditions who may have difficulty cooperating during dental treatment. - **Patients with a strong gag reflex:** N2O/O2 can help suppress the gag reflex, facilitating certain dental procedures. - **Mild to moderate pain control:** N2O/O2 offers some analgesic effect, helping to manage mild to moderate pain during dental procedures. - **Short procedures:** N2O/O2 is ideal for relatively short dental procedures as its effects wear off quickly after administration is stopped.   **Contraindications:**   - **Respiratory conditions:** N2O/O2 should be used with caution in children with respiratory conditions like severe asthma, COPD, or cystic fibrosis. - **Middle ear problems:** N2O/O2 can increase pressure in the middle ear, so it should be avoided in children with recent ear infections or surgery. - **Severe emotional or behavioral problems:** N2O/O2 may not be suitable for children with severe emotional or behavioral problems that require a different sedation approach. - **Pregnancy:** N2O/O2 should be avoided during pregnancy, especially in the first trimester. - **Some medications:** N2O/O2 may interact with certain medications, so it's important to inform the dentist about any medications the child is taking.   **Adverse Effects:**   - **Nausea and vomiting:** These are the most common side effects, but they are usually mild and infrequent. - **Dizziness or lightheadedness:** These can occur but usually subside quickly after the N2O/O2 is discontinued. - **Headache:** This is a less common side effect that may occur after the procedure. - **Diffusion hypoxia:** This is a rare but potential complication that can occur if the N2O/O2 is discontinued abruptly. It can be prevented by administering 100% oxygen for a few minutes after the sedation.   It's important to note that N2O/O2 should only be administered by trained professionals who are experienced in its use with children. The dentist will assess the child's medical history and current condition to determine if N2O/O2 is appropriate and safe. | 1. **Indications**:    - **Anxiety Reduction**: N₂O/O₂ inhalation is effective in reducing anxiety and enhancing communication between the patient and the dental provider.    - **Analgesia**: It provides mild pain relief during dental procedures.    - **Behavior Management**: For patients who need to remain still during treatment. 2. **Contraindications**:    - **Physical and Emotional Development**: Consider the patient’s age and developmental stage.    - **Medical and Dental Histories**: Assess any relevant medical conditions or previous dental experiences.    - **Dental Treatment Needed**: Evaluate the specific procedure and its requirements.    - **Alternative Behavior Guidance Options**: Explore other behavior management techniques.    - **Credentials and Training**: Ensure that the dentist and dental personnel are adequately trained.    - **Equipment and Facilities**: Verify the availability of appropriate equipment.    - **Administration Techniques and Monitoring**: Follow proper protocols.    - [**Occupational Safety**: Consider safety for both patient and dental staff^1^](https://www.aapd.org/research/oral-health-policies--recommendations/use-of-nitrous-oxide-for-pediatric-dental-patients/).   **3. Adverse Effects**:   - - **Nausea and Vomiting**: The most common adverse effect, occurring in approximately 0.5% of patients. [Longer administration, fluctuations in nitrous oxide levels, and increased concentrations may increase the risk^2^](https://www.aapd.org/assets/1/7/G_Nitrous.pdf).   Remember that N₂O/O₂ sedation should be used judiciously, considering the patient’s unique needs and safety. If you have any specific concerns, consult with a pediatric dentist or anesthesiologist. | Ashley P, Anand P, Andersson K. Best clinical practice guidance for conscious sedation of children undergoing dental treatment: an EAPD policy document. Eur Arch Paediatr Dent. 2021 Dec;22(6):989-1002. doi: 10.1007/s40368-021-00660-z. PMID: 34453697 |
|  | **ChatGPT 3.5** | **ChatGPT 4** | **Chat GPT 4o** [**‎**](https://bard.google.com/) | **GEMINI** | **GEMINI ADNANCED** | **COPILOT** | **Evidence (literature)** |
| **8.** What medicaments can be used as capping agents for direct pulp capping in primary teeth, and which one exhibits the best clinical and radiographic success rate? | Direct pulp capping in primary teeth involves placing a medicament directly onto an exposed pulp to promote healing and preserve the vitality of the tooth. Several medicaments can be used as capping agents in this procedure, including:   1. Calcium hydroxide: It has been a traditional choice for direct pulp capping due to its alkaline pH, which promotes dentin bridge formation and pulp tissue repair. 2. Mineral trioxide aggregate (MTA): MTA has gained popularity due to its excellent sealing ability, biocompatibility, and ability to stimulate hard tissue formation. It has shown promising results in direct pulp capping procedures. 3. Biodentine: Biodentine is a bioactive dentin substitute that shares similar properties with MTA. It has been increasingly used in direct pulp capping procedures due to its ease of use and favorable clinical outcomes. 4. TheraCal LC: This light-cured, resin-modified calcium silicate-based material has been developed as an alternative to calcium hydroxide and MTA. It offers similar biocompatibility and sealing properties while eliminating the drawbacks associated with traditional calcium hydroxide formulations. 5. Bioceramics: Various bioceramic materials have emerged as potential alternatives to MTA, offering similar properties in terms of biocompatibility and sealing ability.   Among these options, mineral trioxide aggregate (MTA) has been reported to exhibit the best clinical and radiographic success rates in primary teeth direct pulp capping procedures. Numerous studies have demonstrated its efficacy in promoting dentin bridge formation, preserving pulp vitality, and preventing bacterial microleakage. However, the choice of capping agent may also depend on factors such as operator preference, clinical situation, and availability of materials. It's essential to consider the specific characteristics of each medicament and tailor the treatment plan to the individual patient's needs. | Direct pulp capping in primary teeth involves covering exposed pulp tissue with a dental material to promote healing and maintain pulp vitality. Several materials have been used for this purpose, each with varying degrees of clinical and radiographic success. Here are the common medicaments used for direct pulp capping in primary teeth, along with insights into their effectiveness:  **Common Medicaments for Direct Pulp Capping**   1. **Calcium Hydroxide**: Historically, calcium hydroxide has been the standard for direct pulp capping due to its ability to stimulate dentin formation. However, it does not provide a reliable seal against bacterial penetration and can result in tunnel defects within the newly formed dentin. 2. **Mineral Trioxide Aggregate (MTA)**: MTA exhibits excellent biocompatibility and its ability to set in the presence of moisture makes it suitable for use in environments contaminated with saliva. It promotes high-quality dentinal bridge formation and has low cytotoxicity. MTA tends to show better outcomes in terms of both clinical and radiographic success compared to calcium hydroxide. 3. **Biodentine**: Like MTA, Biodentine is known for its bioactivity and promotes the healing of the pulp. It has similar properties to MTA but sets faster, which can be advantageous in clinical settings. Biodentine has shown promising results in studies, comparable to those of MTA. 4. **Bioactive Glass**: This is a newer material that has been studied for direct pulp capping. It releases calcium and phosphate, which are necessary for hydroxyapatite formation and thus helps in the natural repair process of the pulp. 5. **Resin-modified Glass Ionomer Cement (RMGIC)**: While not as commonly used for direct pulp capping as MTA or calcium hydroxide, RMGIC can also be used due to its fluoride release and relatively good sealing properties.   **Which One Exhibits the Best Clinical and Radiographic Success Rate?**  Recent studies and systematic reviews have generally favored **Mineral Trioxide Aggregate (MTA)** for direct pulp capping in primary teeth due to its superior sealing ability, biocompatibility, and ability to stimulate high-quality dentinal bridge formation. MTA has shown higher rates of pulp survival and fewer instances of pathology in follow-up examinations compared to calcium hydroxide and other materials.  **Biodentine** is also emerging as a strong alternative to MTA, especially when considering its handling characteristics and faster setting time, which can be beneficial in pediatric dentistry.  It is important to select a material based on the specific clinical scenario, taking into account the extent of pulp exposure, the presence of infection, and the overall health of the pulp tissue. Consulting current clinical guidelines and literature can provide further insight into the best practices for direct pulp capping in primary teeth. | To answer this question, let's review the commonly used medicaments for direct pulp capping in primary teeth and evaluate which one shows the best clinical and radiographic success rates:  **Commonly Used Medicaments:**   1. **Calcium Hydroxide (Ca(OH)₂)**:    - **Mechanism**: Promotes the formation of a dentinal bridge and has antibacterial properties.    - **Clinical Performance**: Historically used but has shown variability in success rates due to its potential to cause pulp inflammation and resorption. 2. **Mineral Trioxide Aggregate (MTA)**:    - **Mechanism**: Encourages the formation of hard tissue and is biocompatible with excellent sealing properties.    - **Clinical Performance**: Considered the gold standard for direct pulp capping due to its high success rates in terms of pulpal health and dentinal bridge formation. 3. **Biodentine**:    - **Mechanism**: Similar to MTA but with improved handling properties and faster setting time.    - **Clinical Performance**: Shows promising results comparable to MTA, with good biocompatibility and success in forming a dentinal bridge. 4. **Formocresol**:    - **Mechanism**: Traditionally used as a pulpotomy agent but less commonly used for direct pulp capping due to concerns about toxicity and potential for adverse effects.    - **Clinical Performance**: Not typically recommended for direct pulp capping due to safety concerns. 5. **Calcium Silicate-based Materials**:    - **Mechanism**: Promote hard tissue formation with biocompatible properties similar to MTA.    - **Clinical Performance**: Newer materials that show comparable success rates to MTA and Biodentine.   **Best Clinical and Radiographic Success Rate:**  Among the medicaments listed, **Mineral Trioxide Aggregate (MTA)** has been widely regarded as exhibiting the best clinical and radiographic success rates for direct pulp capping in primary teeth. Studies have consistently demonstrated its effectiveness in maintaining pulp vitality, promoting dentinal bridge formation, and showing favorable clinical outcomes compared to other materials​ ([MDPI](https://www.mdpi.com/2077-0383/11/12/3490))​​ ([JOCPD](https://www.jocpd.com/articles/10.17796/1053-4628-39.4.315))​.  However, newer materials like **Biodentine** are emerging as strong alternatives, offering similar success rates with additional benefits in handling and setting time. Further long-term studies are necessary to fully establish their efficacy compared to MTA. | Direct pulp capping (DPC) aims to preserve the vitality of the dental pulp in primary teeth with minimal exposure. Here's a breakdown of medicaments used for DPC and their success rates:  **Medicaments for Direct Pulp Capping in Primary Teeth:**   - **Calcium Hydroxide (CH):** Traditionally used, CH is biocompatible and stimulates dentin formation. However, it has shown mixed success rates, with studies reporting success rates ranging from 60-80% [1]. - **Mineral Trioxide Aggregate (MTA):** A newer material gaining popularity. MTA exhibits excellent biocompatibility, promotes dentin bridge formation, and has shown promising clinical and radiographic success rates exceeding 90% in some studies [1, 2].   **Best Clinical and Radiographic Success Rate:**  Current evidence suggests **Mineral Trioxide Aggregate (MTA)** offers the **best clinical and radiographic success rate** for direct pulp capping in primary teeth.  Here's why MTA is gaining preference:   - **Superior Biocompatibility:** Less likely to irritate the pulp tissue. - **Dentin Bridge Formation:** Promotes the formation of a hard tissue barrier over the exposure site. - **Sealing Properties:** Effectively seals the pulp, reducing the risk of bacterial infiltration. - **Research Support:** Studies show high success rates compared to traditional calcium hydroxide.   **However, it's important to consider other factors:**   - **Cost:** MTA is generally more expensive than calcium hydroxide. - **Technique Sensitivity:** Proper placement of MTA is crucial for success.   **Ultimately, the dentist will choose the most suitable capping agent based on the specific situation and their experience.** | Several medicaments can be used as capping agents for direct pulp capping in primary teeth:   1. **Calcium Hydroxide:** This has been the traditional material of choice but is now considered less favorable due to its potential to cause internal resorption in primary teeth. 2. **Mineral Trioxide Aggregate (MTA):** This newer material has shown promising results with excellent biocompatibility, good sealing ability, and the potential to promote dentin bridge formation. It comes in various forms like white and grey MTA. 3. **Biodentine:** Another newer material similar to MTA, Biodentine also demonstrates good biocompatibility and sealing ability. It is easier to handle than MTA and sets faster. 4. **Enamel Matrix Derivative (EMD):** While not a direct capping agent, EMD can be used in conjunction with other materials to promote healing and dentin bridge formation. 5. **Calcium Silicate Cements:** These cements, like Biodentine and MTA, are biocompatible and stimulate healing. They have a good track record in pulp capping procedures.   **Which one has the best clinical and radiographic success rate?**  According to current research and clinical studies, Mineral Trioxide Aggregate (MTA) exhibits the best clinical and radiographic success rate for direct pulp capping in primary teeth. It has consistently shown excellent results in terms of:   - **Pulp vitality preservation:** MTA promotes the formation of a dentin bridge, which helps maintain pulp vitality. - **Reduced inflammation:** MTA's biocompatibility and sealing ability help reduce inflammation and promote healing. - **Long-term success:** Clinical studies have shown high success rates with MTA even after several years.   While other materials like Biodentine and calcium silicate cements also show promising results, MTA's long-term track record and consistently high success rates make it the preferred choice for many clinicians.  **Important Note:** The success of direct pulp capping in primary teeth depends on various factors like the size of the exposure, the health of the pulp tissue, and the overall oral hygiene of the child. It's essential to consult with a pediatric dentist to determine the most appropriate treatment option for each individual case. | When it comes to **direct pulp capping (DPC)** in primary teeth, several medicaments have been studied. Let’s explore the options and their clinical and radiographic success rates:   1. **Calcium Hydroxide (CH)**:    - CH has been a traditional choice for DPC.    - **Clinical Success**: While it has been used successfully, its long-term clinical success is somewhat limited.    - [**Radiographic Success**: Radiographic outcomes are generally less favorable compared to other materials^1^](https://pdfs.semanticscholar.org/e0d4/2cec022b2a33bb134b54a8e2d78dbc9f06b7.pdf). 2. **Mineral Trioxide Aggregate (MTA)**:    - MTA is gaining popularity due to its biocompatibility and sealing ability.    - **Clinical Success**: MTA shows excellent clinical outcomes for DPC.    - [**Radiographic Success**: Radiographically, MTA performs well and is often considered superior to CH^2^](https://link.springer.com/article/10.1007/s40368-021-00666-7). 3. **Ferric Sulfate (FS)**:    - FS is another option for DPC.    - **Clinical Success**: It has shown good clinical results.    - [**Radiographic Success**: Radiographically, FS performs well, similar to MTA^2^](https://link.springer.com/article/10.1007/s40368-021-00666-7). 4. **Formocresol (FC)**:    - FC has been used historically but is now less favored due to safety concerns.    - **Clinical Success**: It has shown acceptable clinical outcomes.    - [**Radiographic Success**: Radiographically, FC performs well, similar to MTA and FS^2^](https://link.springer.com/article/10.1007/s40368-021-00666-7).   In summary, MTA, FS, and FC exhibit high clinical and radiographic success rates for DPC in primary teeth. Among these, MTA is often preferred due to its biocompatibility and sealing properties. However, individual patient factors and clinical judgment should guide the choice of medicament. [Always consult with a pediatric dentist for personalized recommendations^2^](https://link.springer.com/article/10.1007/s40368-021-00666-7)[^3^](https://www.aapd.org/globalassets/assets/1/7/g-pulp.pdf). | Duggal M, Gizani S, Albadri S, Krämer N, Stratigaki E, Tong HJ, Seremidi K, Kloukos D, BaniHani A, Santamaría RM, Hu S, Maden M, Amend S, Boutsiouki C, Bekes K, Lygidakis N, Frankenberger R, Monteiro J, Anttonnen V, Leith R, Sobczak M, Rajasekharan S, Parekh S. Best clinical practice guidance for treating deep carious lesions in primary teeth: an EAPD policy document. Eur Arch Paediatr Dent. 2022 Oct;23(5):659-666. doi: 10.1007/s40368-022-00718-6. PMID: 36219336.  Stratigaki E, Tong HJ, Seremidi K, Kloukos D, Duggal M, Gizani S. Contemporary management of deep caries in primary teeth: a systematic review and meta-analysis. Eur Arch Paediatr Dent. 2022 Oct;23(5):695-725. doi: 10.1007/s40368-021-00666-7. PMID: 34981447 |
|  | **ChatGPT 3.5** | **ChatGPT 4** | **Chat GPT 4o** [**‎**](https://bard.google.com/) | **GEMINI** | **GEMINI ADNANCED** | **COPILOT** | **Evidence (literature)** |
| **9.** Silver Diamine Fluoride (SDF) in the Management of Dental Caries Lesions: Which types of patients benefit most from SDF, and what are the criteria for selecting teeth for this treatment? | Silver diamine fluoride (SDF) has shown efficacy in managing dental caries lesions, especially in pediatric patients. Here are the types of pediatric patients who may benefit most from SDF treatment and the criteria for selecting teeth for this approach:  **Types of Pediatric Patients Who Benefit Most:**   1. **Young Children:**    - Preschool-aged children with early childhood caries (ECC) or severe early childhood caries (S-ECC) may benefit from SDF treatment, as it provides a non-invasive and well-tolerated option for caries management.    - Children who are unable to cooperate for traditional restorative procedures due to age or anxiety can benefit from SDF treatment, as it does not require drilling or anesthesia. 2. **High Caries Risk Patients:**    - Pediatric patients with a high risk of dental caries, including those with poor oral hygiene habits, frequent consumption of sugary foods and drinks, or limited access to dental care, may benefit from SDF as a preventive measure to arrest carious lesions and prevent further decay. 3. **Medically Compromised Children:**    - Children with medical conditions or developmental disabilities that make traditional restorative procedures challenging or risky may benefit from SDF treatment as a conservative approach to managing dental caries. 4. **Behaviorally Challenged Children:**    - Pediatric patients with behavioral challenges, such as autism spectrum disorder or attention deficit hyperactivity disorder (ADHD), who may have difficulty tolerating conventional dental treatments can benefit from SDF as a less invasive option.   **Criteria for Selecting Teeth for SDF Treatment:**   1. **Presence of Active Caries Lesions:**    - Teeth with active carious lesions, including cavitated and non-cavitated lesions, are suitable candidates for SDF treatment. 2. **Caries Severity:**    - SDF is effective for managing early to moderate caries lesions. However, extensively decayed teeth may still require traditional restorative treatment. 3. **Location of Lesions:**    - SDF can be applied to carious lesions on any tooth surface, including occlusal, proximal, and smooth surfaces. 4. **Patient Cooperation:**    - Children who may have difficulty cooperating for traditional restorative procedures, such as young children or those with special needs, are suitable candidates for SDF treatment due to its non-invasive nature. 5. **Prognosis of the Tooth:**    - Teeth selected for SDF treatment should have a favorable prognosis for long-term retention, considering factors such as remaining tooth structure, periodontal health, and the patient's ability to maintain oral hygiene. 6. **Patient and Parent Preferences:**    - Patient and parent preferences should be considered when selecting teeth for SDF treatment, as some families may prioritize non-invasive approaches or have concerns about traditional restorative procedures.   By considering these factors, pediatric dentists can identify suitable candidates for SDF treatment and effectively manage dental caries lesions in pediatric patients while minimizing discomfort and preserving tooth structure. | Silver Diamine Fluoride (SDF) is an effective agent used in managing dental caries, particularly in pediatric dentistry. Its ease of application and efficacy in arresting caries make it a valuable choice, especially in patients where traditional dental treatment might be challenging. Here’s an overview of which types of pediatric patients benefit the most from SDF and the criteria for selecting teeth for this treatment:  **Pediatric Patients Who Benefit Most from SDF**   1. **Young Children**: SDF is particularly useful for very young children who are not able to cooperate for traditional dental treatments due to their age. 2. **Patients with Special Needs**: Children with special healthcare needs who may have difficulty tolerating more invasive dental procedures can benefit significantly from SDF due to its non-invasive application. 3. **High Caries Risk Patients**: Children who have a high risk of caries development due to factors like poor oral hygiene, frequent snacking on sugary foods, or a history of early childhood caries (ECC). 4. **Limited Access to Dental Care**: Children who have limited access to dental care due to geographical, financial, or other social barriers can benefit from SDF’s ability to arrest caries with potentially fewer dental visits. 5. **Patients with Caries in Multiple Teeth**: In cases where there are multiple active caries lesions, SDF can be an efficient way to manage the caries and prevent the progression of lesions across multiple visits.   **Criteria for Selecting Teeth for SDF Treatment**   1. **Active Caries Lesions**: SDF is ideal for treating active caries lesions that are not yet involving the pulp. If there is evidence of pulpal involvement, more invasive treatment may be necessary. 2. **Tooth Type and Location**: Primary teeth that are not expected to exfoliate soon are good candidates. SDF can be used on both anterior and posterior teeth, but aesthetic considerations may influence its use on anterior teeth due to staining. 3. **Depth of Caries**: Lesions that are superficial to moderately deep and do not exhibit signs of pulpal exposure or infection are suitable for SDF application. 4. **Patient and Parental Acceptance**: Since SDF causes a permanent black stain on the decayed areas of treated teeth, acceptance of this cosmetic outcome by both the patient and the parents is crucial. 5. **Absence of Pain or Infection**: Teeth that are asymptomatic or mildly symptomatic without signs of infection or abscess are suitable for SDF treatment. If a tooth is causing pain, this might indicate more extensive pulpal involvement requiring alternative treatment.   In using SDF, it is also important to conduct a thorough assessment of the patient’s overall dental and medical history to rule out any contraindications such as known allergies to silver. The decision to use SDF should involve a discussion about the benefits and limitations of the treatment, ensuring informed consent from the caregivers. This non-invasive approach can be a game-changer in managing caries in pediatric populations where conventional treatments pose significant challenges | **Types of Pediatric Patients Who Benefit Most from SDF:**   1. **Very Young Children**: Especially those who are pre-cooperative or unable to undergo conventional restorative treatments. 2. **Medically Compromised or Special Needs Patients**: Children with conditions that make traditional dental treatment challenging. 3. **High Caries Risk**: Patients with multiple carious lesions or a high rate of caries progression. 4. **Financial Constraints**: Families unable to afford traditional restorative treatments may benefit from the low-cost nature of SDF. 5. **Dental Phobia**: Children with significant dental anxiety who might not tolerate conventional dental procedures.   **Criteria for Selecting Teeth for SDF Treatment:**   1. **Active Cavitated Lesions**: SDF is primarily used for arresting active cavitated carious lesions. 2. **Non-Symptomatic Teeth**: Teeth without signs of pulpitis or abscesses are ideal candidates. 3. **Teeth with No Pulpal Involvement**: SDF is not suitable for teeth with deep carious lesions involving the pulp. 4. **Primary Teeth Near Exfoliation**: Particularly useful for primary teeth nearing their natural exfoliation where more invasive treatment is not warranted. 5. **Non-Aesthetic Zones**: Due to the black staining caused by SDF, it is typically used on posterior teeth or areas not visible during a smile.   **Additional Considerations:**   - **Parental Consent**: Parents should be informed and consent to the aesthetic outcomes of SDF treatment. - **Follow-up Care**: Regular follow-up is necessary to monitor the arrested lesions and ensure the effectiveness of the treatment.   Silver Diamine Fluoride (SDF) offers a minimally invasive, cost-effective option for managing dental caries, especially in pediatric patients who may not tolerate traditional dental treatments. It is important to select suitable candidates and provide thorough information to parents about the benefits and limitations of this treatment. | Silver Diamine Fluoride (SDF) is emerging as a valuable tool for managing dental caries lesions in children, particularly for those who might not be ideal candidates for traditional restorative treatments. Here's a breakdown of which pediatric patients benefit most from SDF and the criteria for selecting teeth for treatment:  **Types of Pediatric Patients Who Benefit Most from SDF:**   - **Children with Dental Anxiety:** SDF is a painless topical application, making it suitable for anxious children who might struggle with drilling or injections. - **Very Young Children:** SDF can be a good option for managing cavities in very young children who may not yet have the maturity or ability to cooperate fully for restorative procedures. - **Limited Dental Access:** For children with limited access to dental care or who face financial barriers, SDF offers a cost-effective and less time-consuming way to arrest caries progression. - **Special Needs Children:** Children with special needs who may have difficulty tolerating traditional dental procedures can benefit from SDF's non-invasive approach.   **Criteria for Selecting Teeth for SDF Treatment:**   - **Cavity Type:** SDF is most effective for **incipient caries lesions** (early decay) and **established cavitations** (deeper cavities) in dentin, especially without signs of pulp involvement (inflammation). - **Pain Level:** SDF is not intended for teeth with existing pain or symptoms. - **Restorability:** SDF can be used as a temporary arrest measure before definitive restoration, or for teeth with limited restorability due to extensive decay or crown structure loss.   **Here are some additional points to consider:**   - SDF can cause darkening of the treated tooth, which is a major drawback for some patients. Informed consent discussing this side effect is crucial. - SDF may not be suitable for all children, and a dentist will assess each case individually based on the child's dental needs and overall health.   Overall, SDF offers a valuable and accessible option for managing dental caries in pediatric patients, particularly those facing challenges with traditional restorative treatments. | Silver Diamine Fluoride (SDF) is a non-invasive treatment for dental caries that has gained popularity in pediatric dentistry due to its effectiveness and ease of application. However, it is essential to identify the most suitable candidates and appropriate teeth for SDF treatment to maximize its benefits.  **Pediatric Patients Who Benefit Most from SDF:**   - **High caries risk:** Children with multiple caries lesions, particularly those with special healthcare needs, behavioral management issues, or difficulty accessing dental care, are ideal candidates. SDF can effectively arrest caries progression and reduce the need for invasive treatments. - **Young children:** SDF is a valuable option for very young children who may not tolerate traditional dental procedures. Its quick and painless application makes it a less traumatic experience for them. - **Patients with limited access to care:** In communities with limited access to dental services or where cost is a barrier, SDF can be a valuable tool for managing caries and preventing further tooth decay. - **Patients with dental anxiety or phobia:** SDF's non-invasive nature can be less intimidating for children with dental anxiety, making it easier to provide necessary care.   **Criteria for Selecting Teeth for SDF Treatment:**   - **Cavitated lesions:** SDF is most effective in arresting active cavitated caries lesions in both primary and permanent teeth. - **Accessibility:** The targeted teeth should be easily accessible for SDF application, ensuring proper coverage of the lesion. - **Parental consent:** Informed consent from parents or guardians is crucial before initiating SDF treatment, explaining the benefits, risks, and potential side effects like tooth discoloration. - **Alternative treatment options:** In some cases, SDF may be used as an interim measure before definitive restorative treatment, especially when the child is not yet ready for traditional procedures.   **Additional Considerations:**   - **Esthetics:** SDF causes black staining of the treated areas, which might be a concern for some parents. However, the discoloration is limited to the carious lesion and can be masked with restorative materials later. - **Application frequency:** Multiple applications of SDF might be necessary for optimal results, depending on the severity of the lesion and the child's caries risk. - **Follow-up care:** Regular dental check-ups are crucial to monitor the effectiveness of SDF treatment and assess the need for further intervention.   In conclusion, SDF is a valuable tool in managing dental caries in pediatric patients, particularly those with high caries risk, young children, and those with limited access to care. By selecting appropriate teeth for SDF treatment and considering the individual needs of each child, dentists can utilize this treatment to improve oral health outcomes and prevent further tooth decay. | Let’s explore the use of **Silver Diamine Fluoride (SDF)** in managing dental caries lesions in pediatric patients:   1. **Benefit for Pediatric Patients**:    - **Very Young Children**: SDF can be beneficial for very young children who may have difficulty with other cavity treatments.    - **Persons with Intellectual/Developmental Disabilities**: SDF provides an alternative for those with special needs.    - **Older Adults**: SDF may be preferred by older adults.    - [**Limited Access to Dental Care**: Patients with limited access to dental services can benefit from SDF treatment^1^](http://centerforevidencebasedpolicy.org/wp-content/uploads/2018/06/SDF-Patient-Fact-Sheet-FINAL.pdf). 2. **Criteria for Selecting Teeth for SDF Treatment**:    - **Caries Diagnosis**: SDF should be considered based on a dentist’s diagnosis, including dental radiographs when possible.    - **Extreme Caries Risk**: Patients with extreme caries risk and xerostomia may benefit from more frequent follow-up and reapplication.    - **Non-Restorative Management**: SDF is used as a non-restorative technique to arrest the progression of small cavities and cavity-susceptible areas.    - [**Urgent Referral**: The presence of dental caries indicating SDF use warrants an urgent referral to a dental provider for comprehensive treatment^2^](https://www.aapd.org/globalassets/sdf-guidance-for-physicians_aapd_2023.pdf)[^3^](https://dimensionsofdentalhygiene.com/article/a-practical-guide-to-silver-diamine-fluoride/).   Remember that SDF is not a definitive treatment but can be part of a comprehensive caries management plan. Consult with a dental professional for personalized recommendations | AAPD Guidelines. Chairside Guide: Silver Diamine Fluoride in the Management of Dental Caries Lesions. Pediatr Dent. 2018 Oct 15;40(6):492-517. |
|  | **ChatGPT 3.5** | **ChatGPT 4** | **Chat GPT 4o** [**‎**](https://bard.google.com/) | **GEMINI** | **GEMINI ADNANCED** | **COPILOT** | **Evidence (literature)** |
| **10.** Which materials should be used for Pit and Fissure Sealants in Children's Permanent Molars? | Pit and fissure sealants are resin-based materials applied to the occlusal surfaces of permanent molars to prevent dental caries by sealing off the deep pits and fissures where bacteria and food particles can accumulate. The choice of materials for pit and fissure sealants in children's permanent molars typically includes the following options:   1. **Resin-Based Sealants:**    - Resin-based sealants are the most commonly used materials for pit and fissure sealants due to their excellent sealing ability, durability, and esthetics.    - These sealants are usually composed of a resin monomer (e.g., bisphenol A-glycidyl methacrylate, or BIS-GMA) and filler particles (e.g., silica or glass particles) to provide strength and wear resistance.    - Resin-based sealants can be further classified into two types: conventional (light-cured) sealants and moisture-tolerant (self-cured or light-cured) sealants. 2. **Glass Ionomer Sealants:**    - Glass ionomer sealants are another option for pit and fissure sealants in children's permanent molars.    - These sealants are composed of glass particles suspended in an acidic matrix of polyacrylic acid or similar material.    - Glass ionomer sealants have the advantage of chemical adhesion to tooth structure, which can enhance retention, especially in partially erupted or difficult-to-isolate teeth.    - However, they may be less wear-resistant than resin-based sealants and may require periodic reapplication. 3. **Hybrid Sealants:**    - Hybrid sealants combine features of resin-based and glass ionomer sealants to offer improved properties such as enhanced wear resistance, fluoride release, and adhesion.    - These sealants typically contain a combination of resin monomers and glass ionomer components to capitalize on the benefits of both materials.    - Hybrid sealants may provide a good compromise between the longevity of resin-based sealants and the adhesion of glass ionomer sealants.   When selecting a material for pit and fissure sealants in children's permanent molars, considerations should include the child's caries risk, the condition of the tooth surfaces, the presence of developmental anomalies, and the clinician's preference and experience. Regardless of the material chosen, proper isolation, cleaning, and etching of the tooth surface are essential for ensuring successful sealant placement and long-term retention. Regular follow-up and maintenance visits are also important to monitor the integrity of the sealants and reapply them if necessary. | Pit and fissure sealants are a preventive dental treatment used primarily to prevent dental caries in children and adolescents by sealing the deep grooves and pits in the molars that are prone to decay. The choice of material for pit and fissure sealants depends on various factors including efficacy, retention rates, ease of application, and biocompatibility. Here are the primary materials used for pit and fissure sealants:   1. **Resin-Based Sealants**:    - **Composition**: These sealants are made from a bis-GMA resin or a similar material.    - **Advantages**: They are highly effective due to their ability to bond well with the enamel. Resin-based sealants are durable and can last for many years if properly applied.    - **Application**: Requires a dry working field to adhere properly, which can be challenging in young children or in deep, retentive pits and fissures.    - **Technique Sensitivity**: They are more technique-sensitive because moisture contamination can compromise their adhesion. 2. **Glass Ionomer Sealants**:    - **Composition**: These are made from glass ionomer cements that contain fluoroaluminosilicate glass and an organic acid.    - **Advantages**: Less sensitive to moisture during application, making them easier to use in less controlled environments. They also release fluoride, which can help further protect the teeth from decay.    - **Disadvantages**: Generally, they are less wear-resistant and less retentive than resin-based sealants but have been improving with new formulations.    - **Use**: Often used in situations where maintaining a dry field is difficult or for patients at high risk of caries who would benefit from additional fluoride exposure. 3. **Polyacid-Modified Resin Sealants (Compomers)**:    - **Composition**: These sealants combine properties of glass ionomer and composite resins.    - **Advantages**: They bond well with enamel and release fluoride, though typically less than glass ionomers.    - **Disadvantages**: They require a dry field for application, similar to resin-based sealants, and may not release as much fluoride as traditional glass ionomers. 4. **Resin-Modified Glass Ionomer Sealants**:    - **Composition**: These are a hybrid of resin-based and glass ionomer technologies.    - **Advantages**: They have better esthetic properties and adhesive capabilities than traditional glass ionomers and maintain some fluoride-releasing properties.    - **Application**: Like glass ionomers, they are less sensitive to moisture during application than pure resin-based sealants.   **Choosing the Right Material**  When selecting a material for pit and fissure sealants:   - **Resin-based sealants** are preferred for their durability and efficacy, particularly in controlled clinical settings where moisture control can be maintained. - **Glass ionomer sealants** are a good choice in younger children, less cooperative patients, or situations where moisture control is a concern. They are also beneficial for patients at high risk of caries due to their fluoride release.   The choice between these materials often depends on the specific clinical situation and the dentist's judgment regarding the best fit for the patient's needs and the clinical setting. Regular monitoring and possibly reapplication of sealants are essential to maintain their effectiveness over time. | **Materials for Pit and Fissure Sealants in Children's Permanent Molars**  Pit and fissure sealants are a crucial preventive measure to protect children's permanent molars from dental caries. The choice of material can significantly influence the effectiveness and longevity of the sealant. Here are the commonly used materials for pit and fissure sealants in children's permanent molars:  **1. Resin-Based Sealants**  **Advantages:**   - **Excellent Retention**: Resin-based sealants have superior retention properties compared to other materials. - **Effective Caries Prevention**: They effectively prevent caries by forming a strong barrier against food particles and bacteria. - **Ease of Application**: Available in both light-cured and self-cured forms, making them convenient for different clinical settings.   **Types:**   - **Light-Cured Resin Sealants**: Require a curing light to harden, offering control over working time. - **Self-Cured Resin Sealants**: Harden without the need for a curing light, suitable for situations where light access is challenging.   **Considerations:**   - **Moisture Sensitivity**: Resin-based sealants require a dry field during application to achieve optimal adhesion and longevity. Proper isolation techniques, such as rubber dam usage, are essential.   **2. Glass Ionomer Sealants**  **Advantages:**   - **Fluoride Release**: Glass ionomer sealants release fluoride, which can help in remineralizing adjacent enamel and preventing secondary caries. - **Moisture Tolerance**: They are more tolerant to moisture during application, making them suitable for young children or situations where isolation is difficult.   **Types:**   - **Conventional Glass Ionomer Sealants**: Set through an acid-base reaction and release fluoride over time. - **Resin-Modified Glass Ionomer Sealants**: Combine the benefits of glass ionomer with improved physical properties due to the inclusion of resin. They have better wear resistance and retention than conventional glass ionomer sealants.   **Considerations:**   - **Retention**: Glass ionomer sealants generally have lower retention rates compared to resin-based sealants, so they may need to be checked and reapplied more frequently.   **3. Polyacid-Modified Resin Composites (Compomers)**  **Advantages:**   - **Fluoride Release**: Like glass ionomer sealants, compomers release fluoride, providing an additional caries-preventive effect. - **Improved Mechanical Properties**: They have better mechanical properties than traditional glass ionomer sealants, offering enhanced durability and wear resistance.   **Considerations:**   - **Retention**: Compomers have better retention than glass ionomers but may not match the longevity of pure resin-based sealants.   **Best Practices for Sealant Application**   1. **Proper Tooth Preparation**:    - Clean the tooth surface thoroughly to remove plaque and debris.    - Use an air abrasion or pumice paste to enhance the sealant's bonding surface. 2. **Isolation and Moisture Control**:    - Use rubber dams or cotton rolls to ensure a dry field during the application of resin-based sealants. 3. **Etching**:    - For resin-based sealants, apply an acid etch (usually phosphoric acid) to the enamel to increase retention. 4. **Application Technique**:    - Follow the manufacturer's instructions for the specific sealant material being used.    - Ensure the sealant flows into all pits and fissures, avoiding air bubbles. 5. **Curing**:    - For light-cured sealants, use an appropriate curing light to harden the material fully. 6. **Evaluation and Follow-Up**:    - Check the sealant for retention and integrity during regular dental visits.    - Reapply sealant if necessary to maintain effective protection against caries.   **Summary**   - **Resin-Based Sealants**: Preferred for their excellent retention and caries prevention, but require a dry field during application. - **Glass Ionomer Sealants**: Suitable for situations where moisture control is challenging, with the added benefit of fluoride release. - **Polyacid-Modified Resin Composites (Compomers)**: Offer a balance between fluoride release and improved mechanical properties compared to glass ionomers.   Each material has its advantages, and the choice may depend on the clinical situation, patient cooperation, and the ability to maintain a dry working environment. Regular follow-up is essential to ensure the longevity and effectiveness of the sealants. | There are two main types of materials used for pit and fissure sealants in children's permanent molars:   1. **Resin-based sealants:** These are the most commonly used type and are generally considered the **preferred option**. They offer several advantages:    - **Strong bond:** They form a strong bond to the tooth enamel, which helps them last longer and resist wear.    - **Durability:** They can last for several years with proper care.    - **Easy application:** They are relatively easy and quick to apply. 2. **Glass ionomer sealants:** These are another option, but they have some drawbacks compared to resin-based sealants:    - **Weaker bond:** They may not form as strong a bond to the tooth enamel, which can increase the risk of them breaking or chipping off.    - **Shorter lifespan:** They may not last as long as resin-based sealants.    - **Esthetics:** They may not be as aesthetically pleasing as resin-based sealants, as they can appear slightly opaque.   **Here's a breakdown of the current recommendations:**   - **Resin-based sealants are generally preferred** for pit and fissure sealants in children's permanent molars due to their superior bond strength and durability. - **Glass ionomer sealants might be an alternative option** in some cases, such as for children with very high caries risk or those who have difficulty tolerating the etching procedure required for resin-based sealants. However, dentists may need to monitor these sealants more frequently due to the potential for them to wear down or chip off sooner.   **Additional Considerations:**   - **New developments:** Research is ongoing to develop improved glass ionomer sealants with better bonding properties and wear resistance. - **Dentist's expertise:** The dentist's experience and preference with different materials can also influence the choice of sealant.   **Conclusion:**  When it comes to pit and fissure sealants for children's permanent molars, **resin-based sealants are currently the preferred option** due to their superior performance. However, consulting with a qualified dentist is essential to determine the most suitable material for each individual child based on their specific needs and risk factors. | The most commonly used and recommended materials for pit and fissure sealants in children's permanent molars are:   1. **Resin-based sealants:**These are the most widely used sealants due to their excellent retention, fluoride release, and wear resistance. They are available in various shades to match the tooth color and can be light-cured or self-cured.   Resinbased sealant   1. **Glass ionomer sealants:**These sealants release fluoride, which can help to prevent caries in the surrounding tooth structure. They are also more tolerant of moisture contamination than resin-based sealants, making them easier to place in some cases. However, they have lower retention and wear resistance compared to resin-based sealants.   Glass ionomer sealant   1. **Resin-modified glass ionomer sealants (RMGIs):**These sealants combine the advantages of both resin-based and glass ionomer sealants. They have good retention and fluoride release, and are more tolerant of moisture than resin-based sealants.   Resinmodified glass ionomer sealant  The choice of sealant material depends on various factors, including the child's caries risk, the tooth's morphology, and the clinician's preference.   - **High caries risk:** Resin-based sealants are generally preferred due to their superior retention and wear resistance. - **Moderate caries risk:** RMGIs can be a good option as they combine the advantages of both resin-based and glass ionomer sealants. - **Low caries risk:** Glass ionomer sealants might be considered, especially if moisture control is a concern.   **Important Considerations:**   - Sealants are most effective when placed soon after the tooth erupts, as this is when the risk of caries development is highest. - Proper isolation and meticulous technique are crucial for the success of sealant placement. - Sealants should be checked regularly during dental check-ups and reapplied if necessary.   By using appropriate sealant materials and following recommended practices, dentists can significantly reduce the risk of caries development in children's permanent molars, contributing to their long-term oral health. | Pit-and-fissure sealants are commonly used to prevent and control carious lesions on the occlusal surfaces of primary and permanent molars in children and adolescents. Here are some key recommendations based on evidence-based guidelines:   1. **Effectiveness of Sealants**:    - [A 2016 guideline panel convened by the American Dental Association (ADA) Council on Scientific Affairs (CSA) and the American Academy of Pediatric Dentistry (AAPD) concluded that **sealants are effective** in preventing and arresting pit-and-fissure occlusal carious lesions of primary and permanent molars in children and adolescents compared to the non-use of sealants or use of fluoride varnishes^1^](https://www.ada.org/resources/ada-library/oral-health-topics/dental-sealants)[^2^](https://www.aapd.org/assets/1/7/G_EBD-Sealants1.PDF).    - Sealants can help reduce the risk of cavities by providing a protective barrier on the occlusal surfaces. 2. **Types of Sealant Materials**:    - [The panel recommends that **any of the materials evaluated** (such as resin-based sealants, resin-modified glass ionomer sealants, glass ionomer cements, and polyacid-modified resin sealants) can be used for application in permanent molars with both sound occlusal surfaces and noncavitated occlusal carious lesions in children and adolescents^3^](https://www.guidelinecentral.com/guideline/9944/).    - However, the available evidence does not provide specific recommendations on the relative merits of one type of sealant material over the others. 3. **Clinical Decision-Making**:    - Clinicians are encouraged to discuss this information with patients or parents of patients.    - [Efforts should be directed toward **increasing the use of sealants** on the occlusal surfaces of primary and permanent molars in children and adolescents^2^](https://www.aapd.org/assets/1/7/G_EBD-Sealants1.PDF).   In summary, using pit-and-fissure sealants is an effective preventive measure to maintain oral health in children and adolescents. If you have any specific concerns or questions, I recommend consulting with a dental professional for personalized advice | AAPD Guidelines. Evidence-based Clinical Practice Guideline for the Use of Pit-and-Fissure Sealants Pediatr Dent. 2016 Oct 15;38(5):120-136. |

| **Questions** | **Evidence (literature)** |
| --- | --- |
| 1. When and how should a dentist use local analgesia to control pain in children, adolescents, and medically compromised individuals during the delivery of oral health care? | Kühnisch J, Daubländer M, Klingberg G, Dougall A, Spyridonos Loizides M, Stratigaki E, Amar JL, Anttonen V, Duggal M, Gizani S. Best clinical practice guidance for local analgesia in paediatric dentistry: an EAPD policy document. Eur Arch Paediatr Dent. 2017 Oct;18(5):313-321. doi: 10.1007/s40368-017-0311-y. PMID: 29022286 |
| 2. What is the etiology of molar-incisor-hypomineralization (MIH)? What are the best clinical practices for preventive and treatment options for MIH teeth according to their severity? | Lygidakis NA, Garot E, Somani C, Taylor GD, Rouas P, Wong FSL. Best clinical practice guidance for clinicians dealing with children presenting with molar-incisor-hypomineralisation (MIH): an updated European Academy of Paediatric Dentistry policy document. Eur Arch Paediatr Dent. 2022 Feb;23(1):3-21. doi: 10.1007/s40368-021-00668-5. PMID: 34669177 |
| 3. In which clinical situations in pediatric dentistry might detailed imaging with periapical and bitewing radiographs be beneficial? | Kühnisch J, Anttonen V, Duggal MS, Spyridonos ML, Rajasekharan S, Sobczak M, Stratigaki E, Van Acker JWG, Aps JKM, Horner K, Tsiklakis K. Best clinical practice guidance for prescribing dental radiographs in children and adolescents: an EAPD policy document. Eur Arch Paediatr Dent. 2020 Aug;21(4):375-386. doi: 10.1007/s40368-019-00493-x. PMID: 31768893 |
| 4. What are the recommended features (concentration [ppmF], frequency, and amount) of fluoride toothpaste in the following age groups of children: (1) up to 2 years, (2) 2-6 years, and (3) over 6 years? | Toumba KJ, Twetman S, Splieth C, Parnell C, van Loveren C, Lygidakis NΑ. Guidelines on the use of fluoride for caries prevention in children: an updated EAPD policy document. Eur Arch Paediatr Dent. 2019 Dec;20(6):507-516. doi: 10.1007/s40368-019-00464-2. PMID: 31631242 |
| 5. What is the clinical effectiveness of different restorative materials (amalgam, conventional glass‑ionomer cements, metal-reinforced glass‑ionomer cements, resin-modified glass-ionomer cements, high viscosity glass-ionomer cements, compomer, composite resin) for the class II cavity restorations in a carious primary teeth. | Amend S, Boutsiouki C, Bekes K, Kloukos D, Gizani S, Lygidakis NN, Frankenberger R, Krämer N. Clinical effectiveness of restorative materials for the restoration of carious lesions in pulp treated primary teeth: a systematic review. Eur Arch Paediatr Dent. 2022 Oct;23(5):761-776. doi: 10.1007/s40368-022-00744-4. PMID: 36056991 |
| 6. Treating deep carious lesions in primary teeth using pulpotomy, which medicament is best: Mineral Trioxide Aggregate (MTA), ferric sulfate (FS), formocresol (FC), or calcium hydroxide? What type of restoration should be used after pulpotomy? | Duggal M, Gizani S, Albadri S, Krämer N, Stratigaki E, Tong HJ, Seremidi K, Kloukos D, BaniHani A, Santamaría RM, Hu S, Maden M, Amend S, Boutsiouki C, Bekes K, Lygidakis N, Frankenberger R, Monteiro J, Anttonnen V, Leith R, Sobczak M, Rajasekharan S, Parekh S. Best clinical practice guidance for treating deep carious lesions in primary teeth: an EAPD policy document. Eur Arch Paediatr Dent. 2022 Oct;23(5):659-666. doi: 10.1007/s40368-022-00718-6. PMID: 36219336.  Stratigaki E, Tong HJ, Seremidi K, Kloukos D, Duggal M, Gizani S. Contemporary management of deep caries in primary teeth: a systematic review and meta-analysis. Eur Arch Paediatr Dent. 2022 Oct;23(5):695-725. doi: 10.1007/s40368-021-00666-7. PMID: 34981447 |
| 7. What are the indications, contraindications and adverse effects of using Nitrous oxide/Oxygen sedation in paediatric dental patients | Ashley P, Anand P, Andersson K. Best clinical practice guidance for conscious sedation of children undergoing dental treatment: an EAPD policy document. Eur Arch Paediatr Dent. 2021 Dec;22(6):989-1002. doi: 10.1007/s40368-021-00660-z. PMID: 34453697 |
| 8. What medicaments can be used as capping agents for direct pulp capping in primary teeth, and which one exhibits the best clinical and radiographic success rate? | Duggal M, Gizani S, Albadri S, Krämer N, Stratigaki E, Tong HJ, Seremidi K, Kloukos D, BaniHani A, Santamaría RM, Hu S, Maden M, Amend S, Boutsiouki C, Bekes K, Lygidakis N, Frankenberger R, Monteiro J, Anttonnen V, Leith R, Sobczak M, Rajasekharan S, Parekh S. Best clinical practice guidance for treating deep carious lesions in primary teeth: an EAPD policy document. Eur Arch Paediatr Dent. 2022 Oct;23(5):659-666. doi: 10.1007/s40368-022-00718-6. PMID: 36219336.  Stratigaki E, Tong HJ, Seremidi K, Kloukos D, Duggal M, Gizani S. Contemporary management of deep caries in primary teeth: a systematic review and meta-analysis. Eur Arch Paediatr Dent. 2022 Oct;23(5):695-725. doi: 10.1007/s40368-021-00666-7. PMID: 34981447 |
| 9. Silver Diamine Fluoride (SDF) in the Management of Dental Caries Lesions: Which types of patients benefit most from SDF, and what are the criteria for selecting teeth for this treatment? | AAPD Guidelines. Chairside Guide: Silver Diamine Fluoride in the Management of Dental Caries Lesions. Pediatr Dent. 2018 Oct 15;40(6):492-517. |
| 10. Which materials should be used for Pit and Fissure Sealants in Children's Permanent Molars? | AAPD Guidelines. Evidence-based Clinical Practice Guideline for the Use of Pit-and-Fissure Sealants Pediatr Dent. 2016 Oct 15;38(5):120-136. |
